# Liver X receptor agonists enhance intestinal repair in neonatal piglets with massive bowel resection

**DOI:** 10.1016/j.jlr.2026.101071

**Published:** 2026-05-28

**Authors:** Haixia Feng, Weipeng Wang, Wenjie Wu, Ying Lu, Wei Cai, Yongtao Xiao

**Affiliations:** 1 Department of Pediatric Surgery, Xin Hua Hospital, School of Medicine, Shanghai Jiao Tong University, Shanghai, China; 2 Shanghai Key Laboratory of Pediatric Gastroenterology and Nutrition, Shanghai, China; 3 Division of Pediatric Gastroenterology and Nutrition, Xin Hua Hospital, School of Medicine, Shanghai Jiao Tong University, Shanghai, China; 4 Shanghai Institute for Pediatric Research, Shanghai, China; 5 Department of Pediatric Surgery, Hangzhou Children’s Hospital, Hangzhou, Zhejiang, China

**Keywords:** short bowel syndrome, liver X receptors, intestinal adaption, regeneration

## Abstract

Neonatal short bowel syndrome (SBS) often leads to intestinal failure and dependence on parenteral nutrition (PN). Given the role of liver X receptor (LXR) activation in lipid metabolism and mucosal repair, this study was designed to examine the effects of LXR agonist GW3965 on early adaptive responses in neonatal piglets with SBS. Seven-day-old Bama mini-piglets underwent 75% jejunoileal resection and were randomized into a control group (n = 6) and a GW3965-treated group (n = 4; 2 mg/kg/day administered via jugular vein). The effects of GW3965 on postresection intestinal responses and key molecular pathways were investigated using a combination of in vivo (piglets) and in vitro (human intestinal organoid) models. Administration of the LXR agonist GW3965 promoted significant improvements in gut barrier integrity and mucosal structure in the total parenteral nutrition-supported small-nowel resection piglet model during the 7-day postoperative period. GW3965 significantly increased villus height in the jejunum and ileum (both *P* < 0.05) and crypt depth throughout the remnant intestine and colon (all *P* < 0.05). Consistent with enhanced barrier function, treated piglets exhibited significantly lower serum lipopolysaccharide levels (*P* < 0.01) and elevated expression of tight junction proteins. GW3965 also promoted intestinal epithelial cell proliferation. Complementary in vitro studies confirmed that GW3965 directly stimulated the growth of human intestinal organoids. Notably, GW3965 altered colonic gene expression, upregulating markers typically associated with ileal identity. These findings suggest that targeting the LXR pathway may hold therapeutic potential for augmenting the intestinal regeneration.

Pediatric short bowel syndrome (SBS) represents a perilous malabsorptive disorder, typically emerging from extensive resections of the small intestine necessitated by a plethora of congenital and acquired conditions, including Hirschsprung disease, gastroschisis, intestinal atresia, stenosis, necrotizing enterocolitis (NEC), and volvulus. (1, 2) Pediatric patients afflicted with SBS frequently endure varying degrees of intestinal failure, which often compels them to rely on prolonged periods of parenteral nutrition (PN) support; in certain instances, this dependency may persist throughout their lifetimes. (3) In response to significant intestinal loss, the remaining segments of the intestine embark upon an extraordinary journey of adaptation. This intricate transformation is characterized by remarkable increases in bowel length, diameter, and villus height—an eloquent testament to the body's exceptional capacity for resilience and regeneration. (4, 5, 6, 7, 8) In humans, the process of small-bowel resection (SBR) is intricately intertwined with a rapid adaptive response that culminates in optimal clinical outcomes. Deepening our understanding of intestinal adaptation is imperative for uncovering novel therapeutic interventions that could significantly enhance patient care.

The nuclear receptors known as liver X receptors (LXRs: LXRα, encoded by *NR1H3*, and LXRβ, encoded by *NR1H2*) (9, 10) play an indispensable role in the complex transcriptional regulation of lipid and cholesterol metabolism. (11, 12) LXRs meticulously orchestrate the modulation of a diverse array of genes intricately involved in the uptake, transport, efflux, and excretion of cholesterol. This regulatory mechanism operates with remarkable tissue specificity, dynamically responding to elevated intracellular levels of cholesterol. (12) In the intestinal milieu, a wealth of evidence underscores the physiological significance of the LXR pathway in sterol excretion, thereby illuminating its potential as a compelling target for pharmacological intervention. (13) The LXR agonist GW3965 exhibits a dual regulatory function in both intestinal regeneration and tumorigenesis. Specifically, showing that LXR activation promotes intestinal epithelial repair and reduces colitis. (14) However, these findings have been largely obtained in adult rodents, and whether LXR activation stimulates intestinal regeneration in a neonatal large-animal model of SBS remains unknown. In this investigation, GW3965 was employed to evaluate the impact of LXR activation on intestinal adaptation in SBR piglets. We demonstrated that activation of the LXR pathway improved intestinal adaptation following SBR in piglets by enhancing intestinal regeneration and barrier function, while inhibiting inflammation. Moreover, LXR activation facilitates ileal remodeling of the colon in these SBR-piglets. Collectively, our results indicate that LXR activation promotes intestinal adaptation in SBR piglets, suggesting its potential as a therapeutic strategy for SBS children.

## Materials and methods

### Resection of the small intestine in piglets and the administration of GW3965

One week-old Bama mini-piglets, each weighing approximately 1 kg, were procured from Shanghai Jiagan Biotechnology (Shanghai, China) and subsequently housed at the Experimental Animal Center of Xinhua Hospital. The animals were maintained under a controlled 12-h light/dark cycle. All research protocols involving animal subjects underwent rigorous review and received approval from the Experimental Animal Care and Use Committee of Xinhua Hospital, affiliated with Shanghai Jiao Tong University School of Medicine (XHEC-F-2020-008). The surgical procedures for SBR and the associated postoperative care utilized in this study have been previously documented. (4, 15) The surgical procedures for SBR and the associated postoperative care utilized in this study have been previously documented (4, 15). The piglets were subjected to an 8-h fasting period prior to undergoing SBR operations. Anesthesia was initiated through an intramuscular injection of Zoletil-50 (25 mg/kg; Virbac, France) and subsequently maintained with Isoflurane (Abbott Laboratories). Following this, a significant surgical intervention—a resection encompassing 75% of jejunoileal tissue—was executed; meticulous measurements of the small intestine were taken from the ligament of Treitz to the ileocecal valve. Specifically, this extensive SBR involved excising a segment of small intestine that extended from 30 cm distal to the ligament of Treitz up to 50 cm proximal to the ileocecal valve. The remaining intestinal segments were carefully joined using a single-layer hand-sewn end-to-end jejunoileal anastomosis technique. Postoperatively, analgesia was administered via carprofen injections over a span of 72 h. Furthermore, SBR piglets received PN, adhering rigorously to previously established PN protocols and formulations as documented in our earlier studies. (16, 17) A total of ten Bama mini-piglets were randomly allocated into two groups: one serving as the SBR control group (n = 6) while the other constituted the SBR GW3965 group (n = 4). The latter group received daily injections of GW3965 via jugular vein at a dosage rate of 2 mg/kg body weight for one week. All piglets were humanely sacrificed seven days postsurgery. Body weights were diligently monitored on a daily basis throughout this period. Following sacrifice, measurements for small bowel length—from ligamentum Treitz to ileocecal valve—were repeated meticulously. Samples comprising serum, liver tissue, and intestinal tissues along with specimens from various other organs were collected for subsequent analysis.

### Intestinal morphometrics

Distinct segments of the intestine (Jejunum, Ileum, and Colon) were meticulously preserved in 10% buffered formalin and subsequently subjected to staining with hematoxylin and eosin (H&E). The parameters of villus height, crypt depth, and the ratio of villus height to crypt depth were precisely quantified utilizing QuPath version 0.2. A minimum of ten well-oriented villi and crypts were evaluated across at least three different cross-sections per slide. Average values for both villus height and crypt depth were calculated accordingly. All histological assessments were conducted by experienced pathologists who remained blinded to the experimental groups. Intestinal tissues underwent fixation in 4% paraformaldehyde for a duration of 24 h prior to being sectioned into slices measuring 4 μm for H&E staining. Measurements of villus heights and crypt depths were performed using National Institutes of Health (NIH) Image software (NIH, Bethesda, MD), facilitated by a Nikon microscope (Tokyo, Japan). Villus heights were determined from five well-oriented villi on each slide; five distinct fields per section underwent thorough analysis. Goblet cell counts along with quantification of mucous secretions were accomplished through alcian blue/periodic acid–schiff (AB-PAS) staining techniques. The goblet cell count within each villus was derived from an assessment involving ten well-oriented villi.

### Transmission electron microscopy

Transmission electron microscopy (TEM) analysis was conducted in accordance with established protocols previously delineated (18, 19). In summary, intestinal tissues (ileum and colon) harvested from piglets were meticulously fixed using a 2.5% glutaraldehyde solution at room temperature. Subsequently, the tissues underwent thorough washing before being postfixed in a 1% osmium tetroxide solution within a 0.05 mol/L sodium cacodylate buffer (pH 7.4) at a controlled temperature of 4°C for two hours. Following this step, the specimens were stained with saturated uranyl acetate for an extended duration of three and a half hours at room temperature, dehydrated through graded alcohol solutions, and ultimately embedded in Eponate 12 resin (Ted Pella, Inc., Redding, CA). The resultant sections were then precisely cut utilizing a diamond knife and subjected to staining with a saturated uranyl acetate solution dissolved in 50% ethanol alongside lead citrate. We proceeded to examine and capture images of these sections under the scrutiny of a Philips CM120 transmission electron microscope (Philips Healthcare, Bothell, WA), operating at an accelerating voltage of 80 kV.

### Quantitative real-time polymerase chain reaction (qRT-PCR)

Total RNA was meticulously extracted from intestinal mucosal tissues (jejunum, ileum, and colon) utilizing an RNeasy kit (QIAGEN) in accordance with the manufacturer’s protocol. The quantification of RNA was performed using a NanoDrop spectrophotometer (Applied Biosystems). For reverse transcription, we employed a high capacity cDNA Reverse Transcription Kit (Applied Biosystems), utilizing 2 μg of RNA as the template. Subsequently, real-time PCR reactions were conducted employing a ViiA 7 Real-Time PCR System alongside the PowerUp SYBR Green Master Mix Kit (both from Applied Biosystems). The PCR reactions were incubated in a 384-well plate at 95°C for an initial duration of 10 min, followed by amplification through 40 cycles consisting of denaturation at 95°C for 15 s and annealing/extension at 60°C for one minute. All samples underwent triplicate assays, and data normalization was achieved against the endogenous control *GAPDH*. Relative RNA expression levels were calculated employing the ΔΔCt method. Primers synthesized by Sangon Biotech (Shanghai, China). The primers included: human *GAPDH*: 5′-GTGAACGGATTTGGCCGC-3′ and 5′- AAGGGGTCATTGATGGCGAC-3′; *ABCA1*: 5′- ATGGATCACTGCCCCAGTTC -3′ and 5′- ATGTCCGCGGTGTTCTGTTT -3′; *ABCG1*: 5′- TCCTACGTCAGGTACGGGTT -3′ and 5′- CAGCACGATGAAGTCCAGGT -3′; *TJP1*: 5′- AAGCCCTAAGTTCAATCACAATCT -3′ and 5′- ATCAAACTCAGGAGGCGGC -3′; *EGF*: 5′- GGATGGCCGCTATTCTGTGA -3′ and 5′- CGCCAACGTAGCCAAAAACA -3′; *AREG*: 5′- TTTGGTGAACGATGTGGGGA -3′ and 5′- AGCTGTGAAGCTCATGGCAG -3′; *GAPDH*: 5′- CACATGCGCTCAATCAGTCG -3′ and 5′- TGTATCCGCGGGAAACAGAG -3′; *ABCG5*: 5′- AGCTCAATTTGCTTCCAAGACT -3′ and 5′- TACCCCCAAATGCTAAGGCA -3′; *APOB*: 5′- CCACTGGTCTCCCTCCAAAC -3′ and 5′- AACGCCACACTACCCTCTTG -3′; *FABP6*: 5′- GGCAAGGAGTGCGACATAGA -3′ and 5′- GTAGTTGGGGCTGTTCACCA -3′; *GLUL*: 5′- ATTCCGGAGCGGATGGTTTA -3′ and 5′- TTCAAGTGGGAGCTCGCTGA -3′; *SLC7A9*: 5′- CACTGTGCAGAGACAGGCG -3′ and 5′- GTTCGCTCAGCAAATGCTCTT -3′; *TCN2*: 5′- GCATTTTGGGATGGAAGGC -3′ and 5′- AGGCAAGACAAGTTTCGACG -3′; *FGF19*: 5′- GGGTTTGCTCCGAGTAACGA -3′ and 5′- TATCCGCTAGAAACCGGCAC -3′; *SULT2A1*: 5′- GCCTCATCAGTTCCCACCT -3′ and 5′- GCCTTGGACTTGAAGAAAGC -3′; *TLR4*: 5′- GACAGCAATAGCTTCTCCAGC -3′ and 5′- AAAGGCTCCCAGGGCTAAAC -3′; *NFKB1*: 5′- TTCTGGACCGCTTGGGTAAC -3′ and 5′- CACCGTTGGGGTGGTTGATA -3′; *IL1B*: 5′- CCGAAGAGGGACATGGAGAA -3′ and 5′- AGTTGGGGTACAGGGCAGAC -3′; *IL6*: 5′- TGAACTCCCTCTCCACAAGC -3′ and 5′- GGCAGTAGCCATCACCAGA -3′.

### Western blotting (WB)

For the Western blotting (WB) procedure, approximately 50 mg of intestinal tissue (jejunum, ileum, and colon) was meticulously homogenized in 500 μl of RIPA buffer (Invitrogen, Carlsbad, CA), enriched with a protease inhibitor cocktail (Servicebio). The protein concentration was subsequently quantified using Bicinchoninic acid (BCA) reagent from Pierce Biotechnology (Thermo Fisher Scientific). Following this, equal aliquots of protein were resolved on 10% NuPAGE Bis-Tris gels (Invitrogen) and transferred onto PVDF membranes (MilliporeSigma, Burlington, MA). After blocking the membranes with a solution of 5% nonfat milk to prevent nonspecific binding, they were incubated overnight at 4°C with primary antibodies. Subsequently, the membranes underwent three washes with tris-buffered saline containing 0.1% Polysorbate 20 (TBST), followed by incubation with secondary antibodies. After conducting final washes of the membranes using TBST, we employed an electrochemiluminescence Reagent Kit from Pierce to detect signals. All original WB bands are provided in the supplementary information for reference. The primary antibodies utilized in this study included: β-actin (Cat# 4970S, Cell Signaling Technology), proliferating cell nuclear antigen (Cat# bs-2007R, Bioss), p-STAT3 (Cat# 4113S, Cell Signaling Technology), STAT3 (Cat# 80149-1-RR, Proteintech), Caspase-3 (Cat# 14220S, Cell Signaling Technology), Cleaved Caspase-3 (Cat# 9664, Cell Signaling Technology), lymphatic vessel endothelial hyaluronan receptor 1 (LYVE1) (Cat# bsm-52811R, Bioss), Claudin-1 (Cat# 13255P, Cell Signaling Technology), Occludin (Cat# GB15149, Servicebio), LXR (Cat# 14351-1-AP, Proteintech), P-AKT (Cat# 4060, Cell Signaling Technology), AKT (Cat# GB15689, Servicebio), and CD31 (Cat# GB11063, Servicebio).

### Immunohistochemistry (IHC) staining

Immunohistochemistry (IHC) was meticulously conducted on intestinal sections (jejunum, ileum, and colon) obtained from piglets in the SRR control group (n = 4) and the SBR GW3965 group (n = 4), following previously established protocols (20). In brief, the slides underwent a series of treatments involving xylene and progressively decreasing concentrations of ethanol. To inhibit endogenous peroxidases, a solution of 0.3% H_2_O_2_ was applied for a duration of 10 min at room temperature. Following antigen retrieval, blocking was executed using 5% bovine serum albumin for 30 min at room temperature. The antibodies were then introduced at their optimal concentrations and allowed to incubate overnight in a humidified chamber at 4°C. Subsequent to this incubation period, the slides were thoroughly rinsed with PBS before being treated with the appropriate secondary antibody for one hour at room temperature. After another round of PBS rinsing, counterstaining was performed using hematoxylin. Five randomly selected high-power microscopic fields were analyzed per sample. Image analysis was carried out utilizing Image Pro Plus software (Media Cybernetics, Rockville, MD). A comprehensive list of the primary antibodies included: LXRα (Cat# 14351-1-AP, Proteintech), zonula occludens-1 (ZO-1) (Cat# GB111981, Servicebio), Cleaved Caspase-3 (Cat# GB111981, Servicebio), Ki-67 (GB151499), and I-FABP (Cat# GB115584, Servicebio).

### Intestinal organoids

Fresh ileal tissues were obtained from a patient (male, 5 months) with NEC under operation. The patients’ guardian provided written informed consent. The institutional ethics committee of Xinhua Hospital affiliated to Shanghai Jiao Tong University School of Medicine approved this study (XHEC-D-2022-030). The study was conducted in accordance with the Declaration of Helsinki. The surgical samples were meticulously minced into fragments measuring less than 1 mm^3^ and subsequently incubated in an ice-cold soaking buffer (DPBS enriched with 30 mM EDTA and 1.5 mM DTT) for a duration of 20 min. The tissue fragments were then transferred to DPBS augmented with a twofold concentration of Antibiotic-Antimycotic, followed by dissociation through vertical shaking to liberate the crypts. The resultant suspension was carefully filtered sequentially through a 70 μm nylon mesh to eliminate debris, after which it was treated with 1 × RBC lysis buffer to eradicate erythrocytes. Crypts were pelleted via centrifugation at 200 × *g* for 10 min at a temperature of 4°C, resuspended in cold DMEM supplemented with 10% BSA, and quantified accordingly. Crypts (300–500 per well) were suspended within Matrigel™ at a ratio of one part crypt suspension to two parts Matrigel. Aliquots of this crypt-Matrigel mixture (50 μl each) were plated onto prewarmed 24-well plates and allowed to polymerize at a controlled temperature of 37°C for approximately 20 min. Once polymerized, the Matrigel domes were overlaid with basic culture medium (ENR) containing advanced DMEM/F12, 1× penicillin–streptomycin, 1× GlutaMAX (Thermo Fisher Scientific), 10 mM Hepes (Thermo Fisher Scientific), 1× B27 (Life Technologies), 1× N2 (Life Technologies), and 50 ng ml−1 of murine recombinant epidermal growth factor (EGF, PeproTech), 200 ng ml−1 recombinant R-spondin-1 (MCE) and 100 ng ml^−1^ recombinant murine Noggin (Peprotech). For experiments medium NR, the same medium as above with the exception of EGF was prepared. NR plus amphiregulin (AREG) or epiregulin (EREG) was prepared with supplemented 50 ng ml^−1^ AREG (MCE) or 50 ng ml^−1^ EREG (MCE). GW3965 (1.5 μM, MCE) was added above basic or experimental medium for 3–5 days. After treatments, the organoids were collected and fixed in 4% paraformaldehyde, and then stained with E-cadherin (Alexa Fluo-488 conjugated E-cadherin, BD Biosciences), Phalloidin (red fluorescent dye tetramethylrhodamine, Thermo Fisher Scientific) and counterstain with DAPI (Servicebio).

### Endotoxin analysis

The determination of endotoxin levels was conducted through the assessment of lipopolysaccharide (LPS) concentrations in serum. Following the collection of blood from piglets, samples were subjected to centrifugation at 3,000 *g* for a duration of 15 min. Subsequently, a Chromogenic LAL Endotoxin Assay Kit (C0276S, Beyotime Biotechnology) was employed in accordance with the manufacturer’s guidelines.

### Statistics and reproducibility

Comprehensive numerical source data for all graphical representations are meticulously detailed in the Methods section and accompanying figure legends. Statistical analyses were conducted utilizing GraphPad Prism 8 Software (GraphPad, San Diego, CA), employing an unpaired Student’s *t* test or one-way analysis of variance to facilitate comparisons among distinct groups. For datasets exhibiting non-normal distribution characteristics, the Mann–Whitney U test or Kruskal–Wallis test was employed accordingly. Each mouse was evaluated as an independent sample. The representative data presented herein are expressed as the mean ± SD. *P* values less than 0.05 were deemed statistically significant, with significance levels further delineated as follows: ∗∗*P* < 0.01, ∗∗∗*P* < 0.001, ∗∗∗∗*P* < 0.0001.

## Results

### LXR activation significantly mitigates intestinal injuries and alleviates inflammation in neonatal SBR piglets

**Fig. 1:**
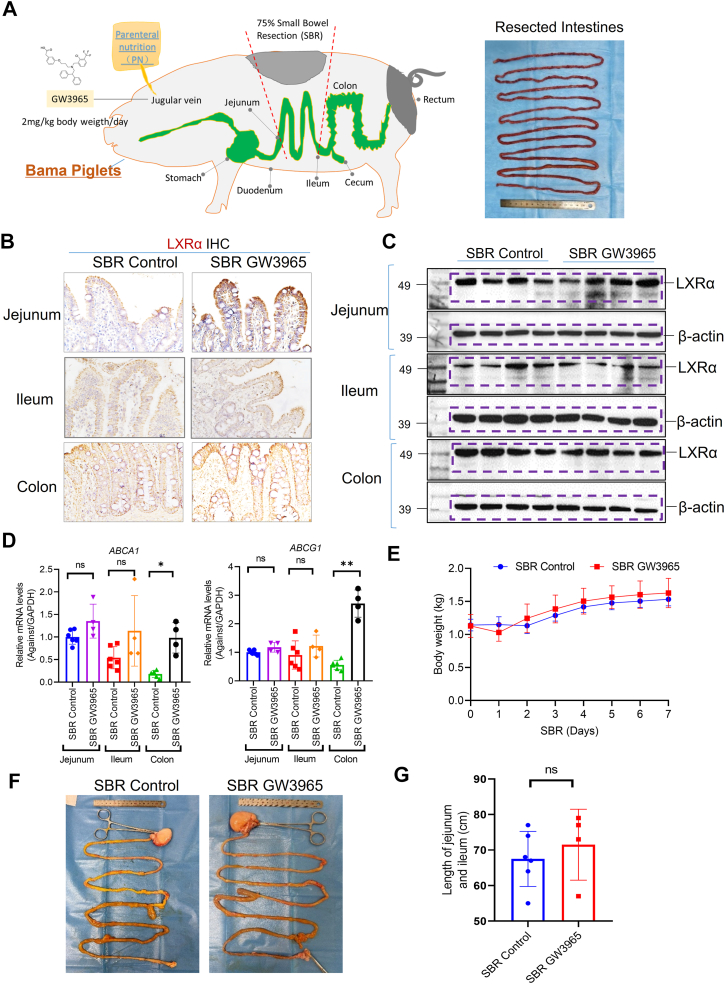
The phenotypes of small-bowel resection (SBR) piglets are altered by LXR activation. A: Schematic of the small-bowel resection (SBR) in Bama mini-piglets. The piglets were received both SBR surgery and total parenteral nutrition (PN). GW3965-treated piglets were daily injected of GW3965 via jugular vein at a dosage rate of 2 mg/kg body weight for one week. B: Representative immunohistochemistry (IHC) images of LXRα in mucosa of jejunum, ileum, and colon from SBR control and SBR GW3965 piglets (each group, n = 4). C: Western blotting (WB) analysis for LXRα in mucosa of jejunum, ileum, and colon from SBR control and SBR GW3965 piglets (each group, n = 4). D: Quantitative real-time polymerase chain reaction (qRT-PCR) analysis was performed to determine the levels of ATP-binding cassette transporter-A1(*ABCA1*) and ATP-binding cassette G1 (*ABCG1*) in both SBR control (n = 6) and SBR GW3965 piglets (n = 4). E: Comparing body weight monitoring daily between SBR control and SBR GW3965 piglets. F: Representative images of small intestines from SBR control and SBR GW3965 piglets after sacrifice. G: The statistical length of jejunum plus ileum in SBR control (n = 6) and SBR GW3965 piglets (n = 4). Unpaired two-tailed Student’s *t* test with or without Welch’s correction analysis for (D and G). Statistical significance: ns, not significant, ∗*P* < 0.05, ∗∗*P* < 0.01.

**Fig. 2:**
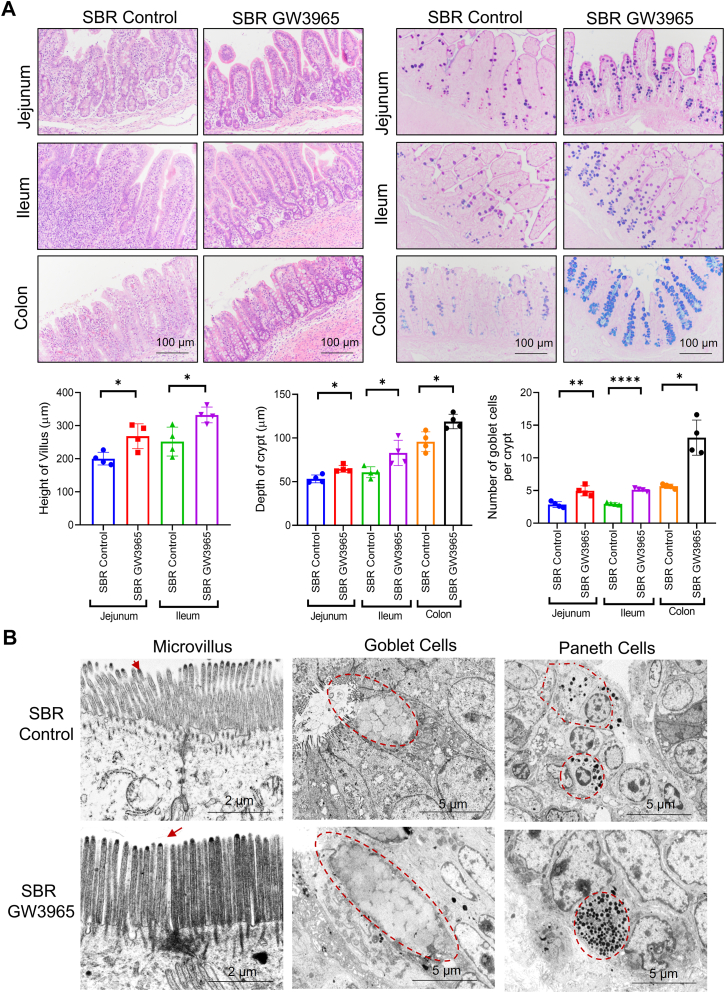
LXR activation improves pathological changes in small-bowel resection (SBR) piglets. A: Representative images of hematoxylin and eosin (H&E) staining and alcian blue/periodic acid schiff (AB-PAS) staining in jejunum, ileum, and colon from both SBR control and SBR GW3965 piglets (each group, n = 4). Quantification of villus height, crypt depth, and goblet cells number per crypt in both two groups (each group, n = 4). B: Transmission electron microscopy (TEM) analysis in the ileums of both SBR control and SBR GW3965 piglets (each group, n = 2). Unpaired two-tailed Student’s *t* test with or without Welch’s correction analysis for (A). Statistical significance: ns, not significant, ∗*P* < 0.05, ∗∗*P* < 0.01, ∗∗∗∗*P* < 0.0001. LXR, liver X receptor.

**Fig. 3:**
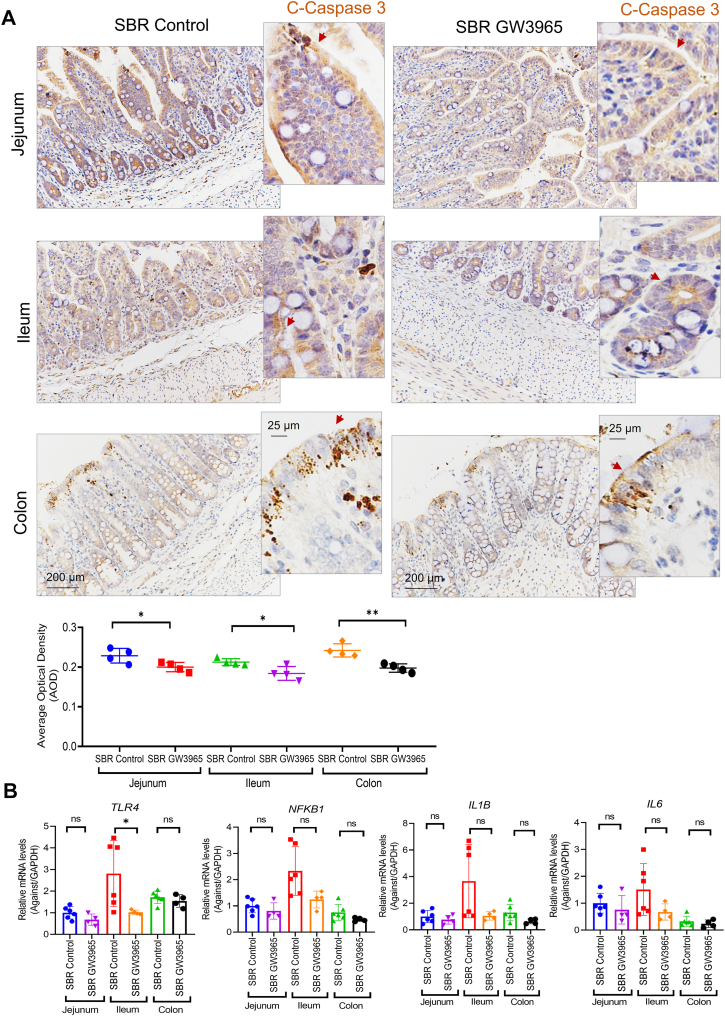
LXR activation inhibits apoptosis and inflammation in small-bowel resection (SBR) piglets. A: Representative immunohistochemistry (IHC) images of cleaved Caspase 3 (C-Caspase 3) in mucosa of jejunum, ileum, and colon from SBR control and SBR GW3965 piglets. Qualification of C-Caspase 3 IHC (each group, n = 4). B: Quantitative real-time polymerase chain reaction (qRT-PCR) analysis was performed on the jejunum, ileum, and colon of SBR control (n = 6) and SBR GW3965 piglets (n = 4) and determined the expression of toll-like receptor 4 (*TLR4*), nitric oxide synthase 2 (*NOS2)*, nuclear factor kappa-light-chain-enhancer of activated B cells 1 (*NFKB1*), interleukin 1 beta (*IL1B*), and interleukin 6 (*IL6)*. The genes were calculated against glyceraldehyde-3-phosphate dehydrogenase (*GAPDH*). Unpaired two-tailed Student’s *t* test with or without Welch’s correction analysis for (A and B). Statistical significance: ns, not significant, ∗*P* < 0.05, ∗∗*P* < 0.01. LXR, liver X receptor.

**Fig. 4:**
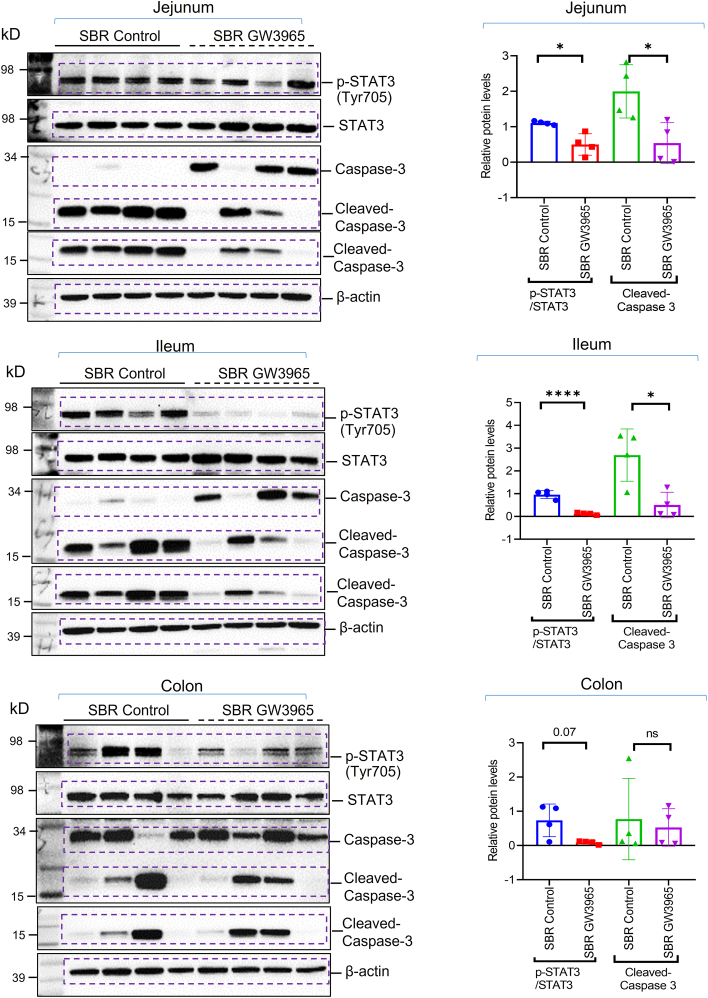
LXR activation suppresses STAT3 phosphorylation and Caspase 3 cleaved in small-bowel resection (SBR) piglets. Western blotting (WB) analysis for phosphorylation of signal transducer and activator of Ttranscription 3 (p-STAT3), STAT3, cleaved Caspase 3, and Caspase 3 in mucosa of jejunum, ileum, and colon from SBR control and SBR GW3965 piglets (each group, n = 4). Quantification of these proteins levels in two groups. Unpaired two-tailed Student’s *t* test with or without Welch’s correction analysis. Statistical significance: ns, not significant, ∗*P* < 0.05, ∗∗∗∗*P* <0.0001. LXR, liver X receptor; p-STAT3, phosphorylated STAT3; STAT3, signal transducer and activator or transcription 3.

In order to elucidate the effects of LXR activation on intestinal adaptation, we initially established a SBR model involving 75% intestinal resection in neonatal piglets (Bama mini piglets, approximately 7 days old). Following the surgical procedure, all piglets commenced PN, wherein four SBR piglets were administered an LXR agonist (GW3965, at a dosage of 2 mg/kg/body weight/day), forming the SBR GW3965 group. Meanwhile, six SBR piglets receiving PN served as the control group for comparison (Fig. 1A). After one week, all piglets were sacrificed, and intestinal tissues were collected for further analysis. Both IHC staining and WB assays demonstrated that LXRα proteins were expressed in the mucosa of the jejunum, ileum, and colon; however, their expression did not show significant alterations following administration of GW3965. (Fig. 1B, C). As anticipated, the quantitative reverse transcription polymerase chain reaction (qRT-PCR) analysis revealed that the LXR target genes *ABCA1* and *ABCG1* were significantly induced in the intestinal mucosa of piglets treated with GW3965, compared to those in SBR control groups. (Fig. 1D). The body weight monitoring indicated that the administration of GW3965 resulted in a slight increase in body growth; however, these changes did not achieve statistically significant levels (Fig. 1E). Similarly, the growth of the small intestines in GW3965-treated piglets showed a nonsignificant increase compared to that in control piglets (Fig. 1F, G). Intestinal mucosal morphometric analysis utilizing H&E staining revealed that GW3965-treated SBR piglets exhibited significantly longer villus height and greater crypt depth compared to SBR control piglets (Fig. 2A, B).AB-PAS staining demonstrated a significant increase in the number of goblet cells in both the small intestines and colons of GW3965-treated SBR piglets relative to those of SBR control piglets (Fig. 2A, B). TEM analysis uncovered irregular microvilli distribution, shorter microvilli, impaired mucin production in goblet cells, and abnormal eosinophilic granules in Paneth cells among SBR piglets; these abnormalities were ameliorated by GW3965 treatment (Fig. 2A, B). As illustrated in Figure 3A, there was a notable reduction in cleaved caspase-3-positive staining cells within the intestinal mucosa of GW3965-treated SBR piglets compared to their untreated counterparts (Fig. 3A). qRT-PCR assays indicated that proinflammatory genes such as *TLR4*, *NFKB1*, *IL1B*, and *IL6* were significantly inhibited by GW3965 treatment when contrasted with SBR control piglets (Fig. 3B). Furthermore, WB analysis corroborated the inhibitory effects of GW3965 on proapoptotic proteins like cleaved caspase-3 as well as on the proinflammatory factor phosphorylated STAT3 among SBR piglets treated with this compound (Fig. 4).

### LXR activation enhances intestinal barrier function in neonatal SBR piglets

**Fig. 5:**
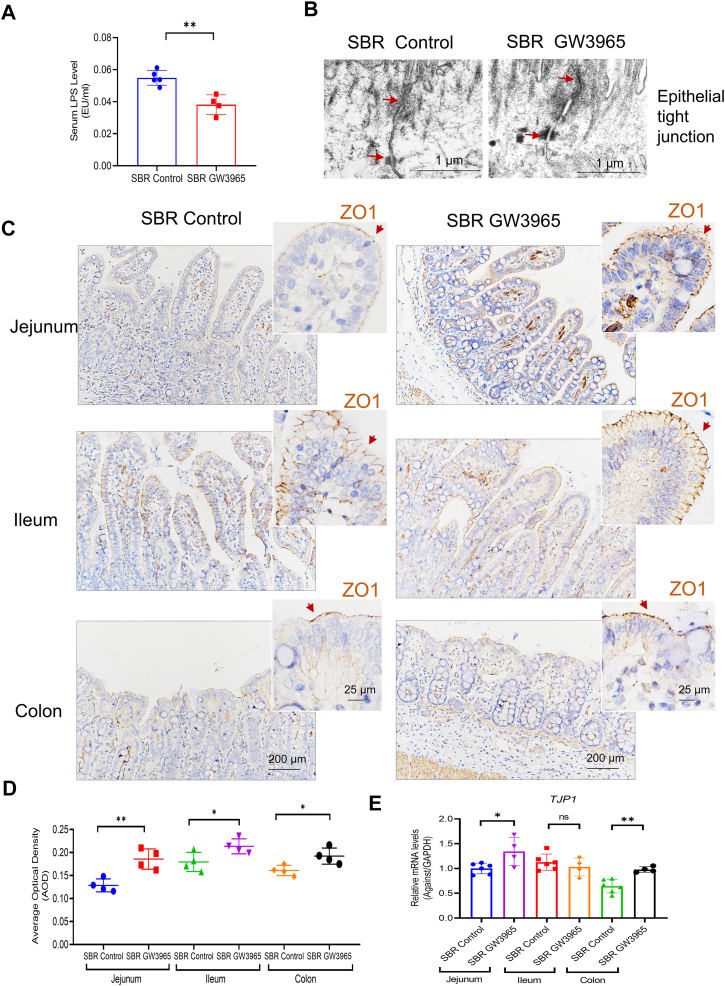
LXR activation increases epithelial tight junction in small-bowel resection (SBR) piglets. A: The serum levels of lipopolysaccharide (LPS) in SBR control (n = 5) and SBR GW3965 piglets (n = 4). B: Transmission electron microscopy (TEM) analysis of tight junctions in the ileums of both SBR control and SBR GW3965 piglets. C:) Representative immunohistochemistry (IHC) images of ZO-1 in mucosa of jejunum, ileum, and colon from SBR control and SBR GW3965 piglets. D: Qualification of ZO-1 IHC in panel (C) (each group, n = 4). E: Quantitative real-time polymerase chain reaction (qRT-PCR) analysis was performed to determine the levels of ZO-1 gene *TJP1* in intestinal mucosa of both SBR control (n = 6) and SBR GW3965 piglets (n = 4). Unpaired two-tailed Student’s *t* test with or without Welch’s correction analysis for (A, D, and E). Statistical significance: ns, not significant, ∗*P* < 0.05, ∗∗*P* < 0.01. LXR, liver X receptor; ZO-1, zonula occludens-1.

**Fig. 6:**
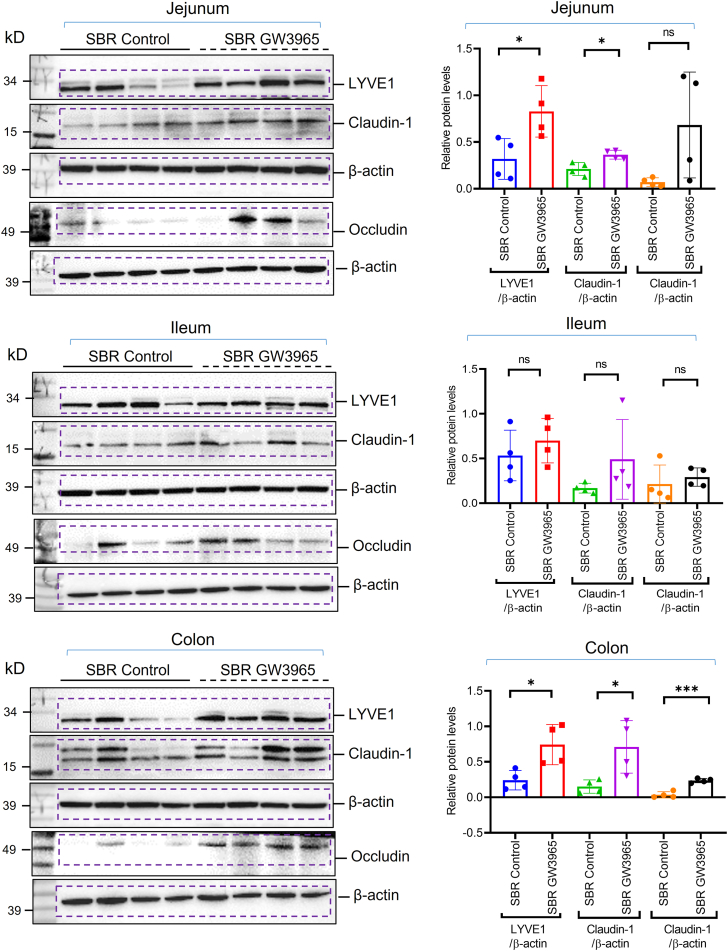
LXR activation enhances epithelial tight junction’s proteins and lymphatic vessel in small-bowel resection (SBR) piglets. Western blotting (WB) analysis for lymphatic vessel endothelial hyaluronan receptor 1 (LYVE1), Occludin and Claudin-1 in mucosa of jejunum, ileum, and colon from SBR control and SBR GW3965 piglets (each group, n = 4). Quantification of these proteins levels in two groups. Unpaired two-tailed Student’s *t* test with or without Welch’s correction analysis. Statistical significance: ns, not significant, ∗*P* < 0.05, ∗∗∗*P* < 0.001. LXR, liver X receptor.

The integrity of the intestinal epithelial barrier in SBR piglets was evaluated by measuring serum levels of LPS. As illustrated in Figure 5A, GW3965-treated SBR piglets exhibited significantly lower serum LPS concentrations compared to those observed in the SBR control group. (Fig. 5A). TEM analysis revealed that GW3965 treatment ameliorated the damage to intercellular junctions among intestinal epithelial cells in SBR piglets (Fig. 5B). IHC staining demonstrated a significant elevation in the expression of the tight junction protein ZO-1 in the intestinal epithelial cells of piglets treated with GW3965, compared to those in SBR control piglets (Fig. 5C, D). Consistently, qRT-PCR analysis indicated that GW3965 treatment increased ZO-1 gene expression, particularly in colonic mucosa (Fig. 5E). WB analysis showed an increase in the expression levels of key tight junction proteins Occludin and Claudin-1, as well as the lymphatic vessel marker LYVE1, within both small intestine and colon tissues from GW3965-treated piglets relative to those derived from SBR control piglets (Fig. 6).

### LXR activation promotes intestinal regeneration

**Fig. 7:**
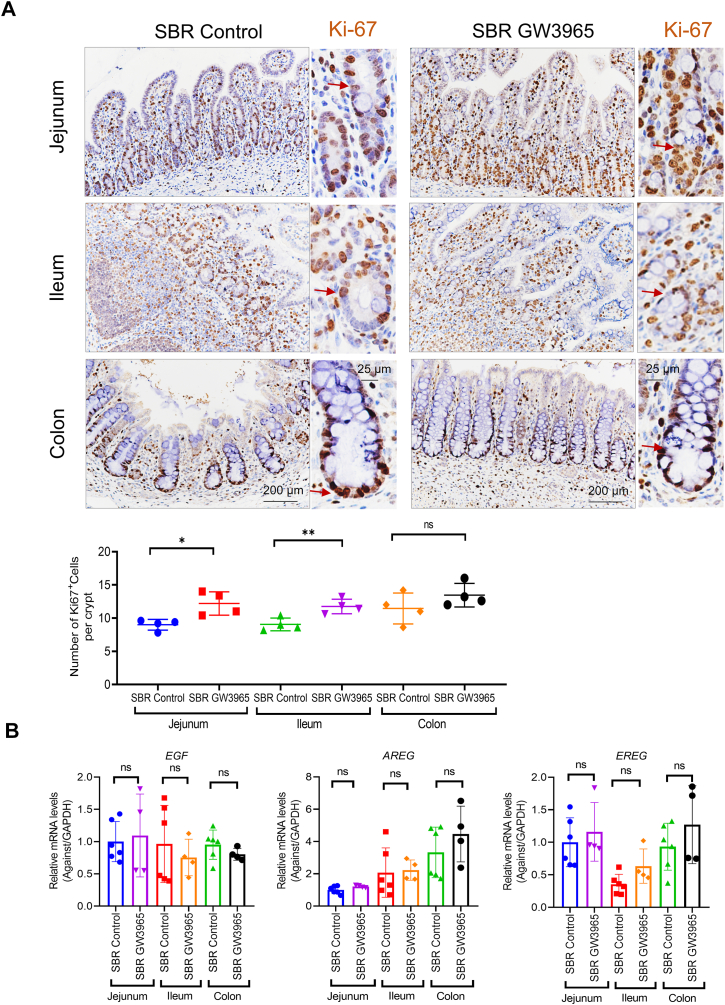
LXR activation promotes intestinal regeneration in small-bowel resection (SBR) piglets. A: Representative immunohistochemistry (IHC) images of proliferative maker Ki-67 in mucosa of jejunum, ileum, and colon from SBR control and SBR GW3965 piglets. Qualification of Ki-67 IHC (each group, n = 4). B: Quantitative real-time polymerase chain reaction (qRT-PCR) analysis was performed on the jejunum, ileum, and colon of SBR control (n = 6) and SBR GW3965 piglets (n = 4) and determined the expression of epidermal growth factor (*EGF*), amphiregulin (*AREG*), and epiregulin (*EREG*). The genes were calculated against glyceraldehyde-3-phosphate dehydrogenase (*GAPDH*). Unpaired two-tailed Student’s *t* test with or without Welch’s correction analysis for (A and B). Statistical significance: ns, not significant, ∗*P* < 0.05, ∗∗*P* < 0.01. LXR, liver X receptor.

**Fig. 8:**
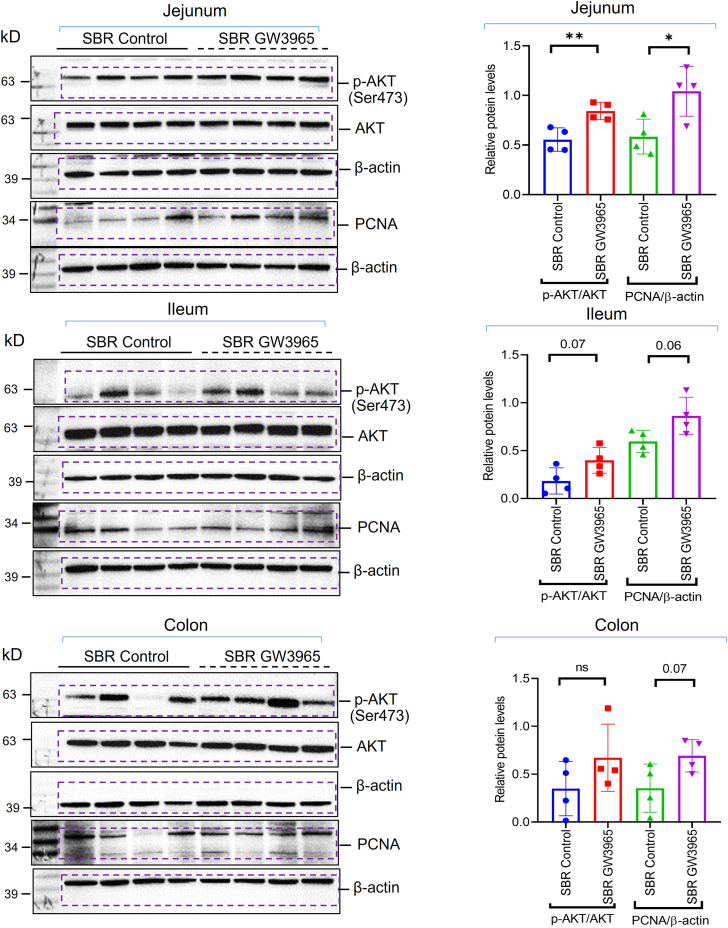
LXR activation enhances AKT phosphorylation and PCNA expression in small-bowel resection (SBR) piglets. Western blotting (WB) analysis for phosphorylated protein kinase B (p-AKT), AKT, and proliferating cell nuclear antigen (PCNA) in mucosa of jejunum, ileum, and colon from SBR control and SBR GW3965 piglets (each group, n = 4). Quantification of these proteins levels in two groups. Unpaired two-tailed Student’s *t* test with or without Welch’s correction analysis. Statistical significance: ns, not significant, ∗*P* < 0.05, ∗∗*P* < 0.01. AKT, protein kinase B; LXR, liver X receptor.

**Fig. 9:**
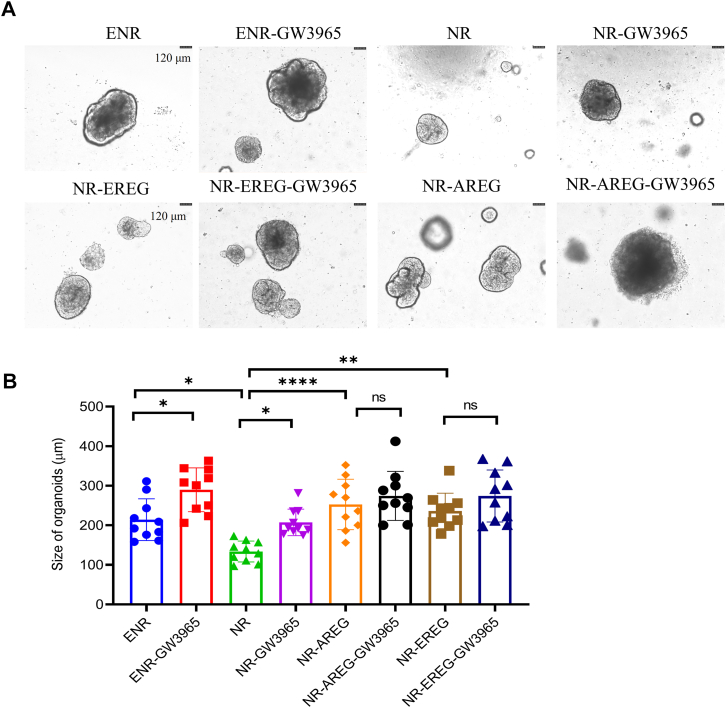
LXR activation with GW3965 promotes growth of human intestinal organoids. A: Representative size and morphology of culture organoids from ileal crypts from a patient with necrotizing enterocolitis (NEC). The organoids were cultured under different condition with ENR, NR, NR-AREG, and NR-EREG in the presence or absence of GW3965 treatments. B: Quantification for the size of organoids. Ordinary One-way ANOVA analysis for (B). ns, not significant, ∗*P* < 0.05, ∗∗*P* < 0.01, ∗∗∗∗*P* < 0.0001; E, EGF, epidermal growth factor; R, RSPO1, R-spondin-1; N, Noggin; AREG, amphiregulin; EREG, epiregulin; LXR, liver X receptor.

**Fig. 10:**
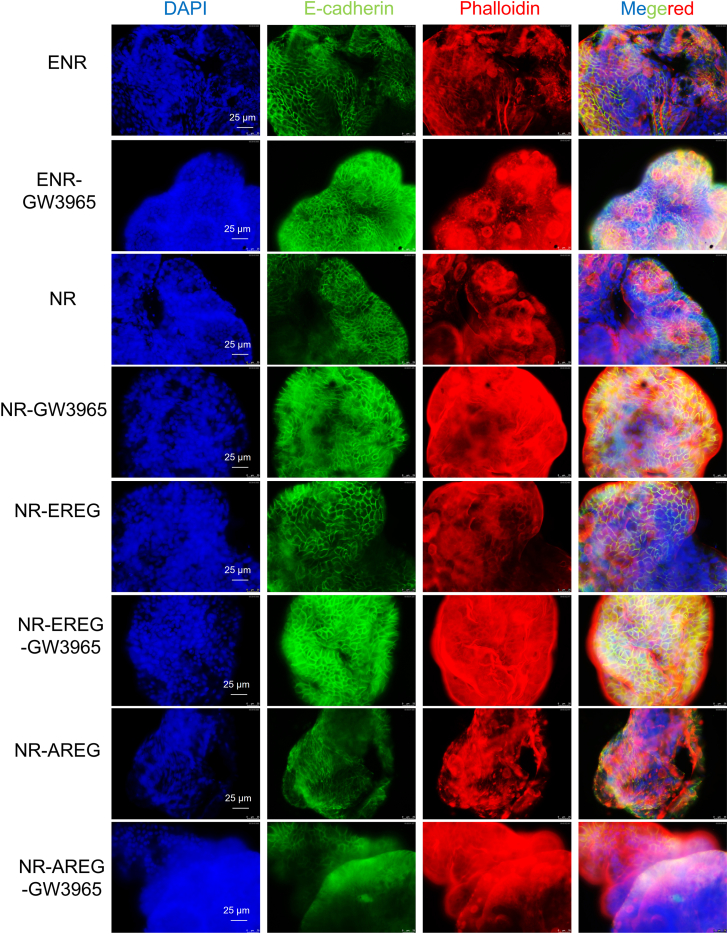
LXR activation with GW3965 enhances integrity of organoids. Representative images of immunofluorescence (IF) staining for E-cadherin (*green*), Phalloidin (*red*), and DAPI (*blue*) for organoids under different condition with ENR, NR, NR-AREG, and NR-EREG in the presence or absence of GW3965 treatments. E, EGF, epidermal growth factor; R, RSPO1, R-spondin-1; N, Noggin; AREG, amphiregulin; EREG, epiregulin; LXR, liver X receptor.

To evaluate whether LXR activation enhances intestinal regeneration in SBR piglets, we first analyzed crypt cell proliferation using the proliferative marker Ki-67 staining. We observed a comparable increase in the number of Ki-67-positive cells in the crypts of GW3965-treated piglets compared to that of SBR controls (Fig. 7). In addition, we noted that LXR activation through GW3965 treatment led to a modest increase in transcript levels of EGF-family ligands *AREG* and *EREG*, but not *EGF* mRNA (Fig. 7). WB analysis indicated GW3965 treatment not only enhanced AKT signaling—an essential pathway for cellular growth—within the mucosa but also elevated the expression levels of the cell proliferation marker proliferating cell nuclear antigen protein (Fig. 8). To functionally ascertain whether LXR activation bolstered intestinal regeneration by priming epithelial tissues, we conducted an intestinal organoid culture utilizing neonatal ileal crypts from a patient with NEC, subsequently stimulating them with GW3965 in vitro. Given that our standard organoid culture medium comprises EGF—a critical component alongside noggin and R-spondin—we observed that LXR activation significantly promoted organoid growth within this conventional culture milieu (Fig. 9). This led us to hypothesize that LXR activation might compensate for any deficiency arising from EGF removal from the culture medium. Indeed, our findings confirmed that LXR activation resulted in markedly enhanced regenerative growth in organoids cultured under NR conditions (noggin and R-spondin; Fig. 9). Subsequently, to explore potential roles played by two additional EGF ligands, AREG and EREG—which were stimulated by GW3965 within the intestinal mucosa—in mediating proregenerative growth in organoids cultivated under NR conditions, we demonstrated that both AREG and EREG substantially augmented organoid development (Fig. 9). Notably, their combination with GW3965 yielded even greater proregenerative effects (Fig. 9). Moreover, we further discerned enhancements in both differentiation and cytoskeletal integrity of intestinal organoids upon introduction of GW3965 into the culture environment (Fig. 10).

### LXR activation enhances ileal remodeling of colon in neonatal SBR piglets

**Fig. 11:**
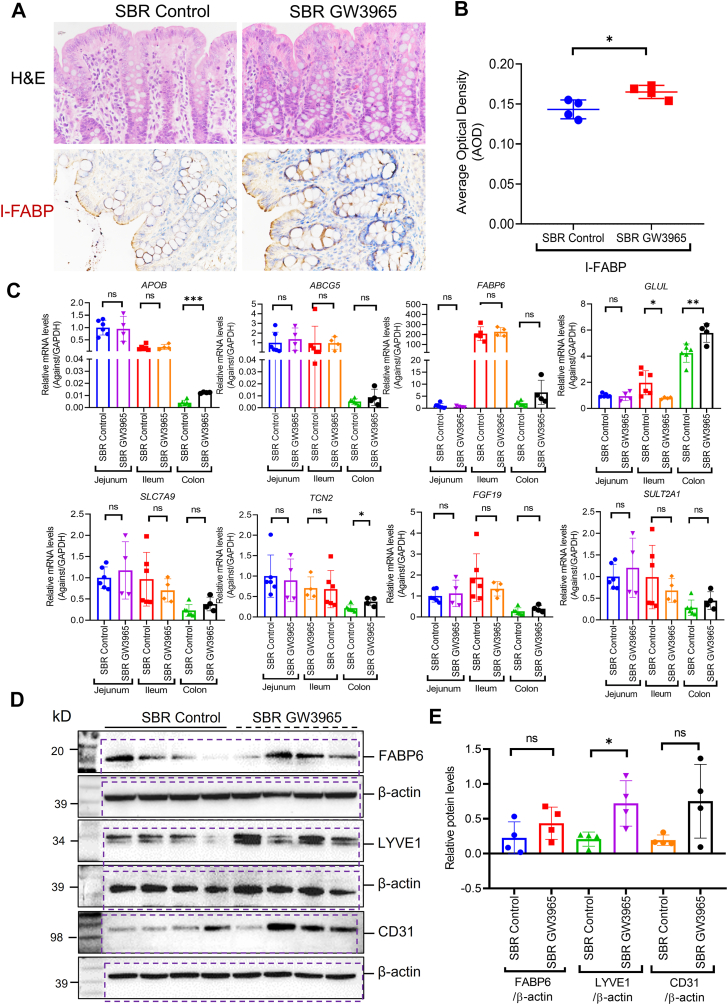
LXR activation promotes ileal remodeling of colon in neonatal SBR-piglets. A: Representative images of hematoxylin and eosin (H&E) staining and immunohistochemistry (IHC) images of I-FABP in mucosa of colon from SBR control and SBR GW3965 piglets. B: Qualification of I-FABP IHC in panel (A). C: Quantitative real-time polymerase chain reaction (qRT-PCR) analysis was performed on the jejunum, ileum, and colon of SBR control (n = 6) and SBR GW3965 piglets (n = 4) and determined the expression of genes of lipid (*ABCG5*, *FABP6*), amino acid (*SLC7A9*, *GLUL*), bile salt (*FGF19*, *SULT2A1*), and vitamin (*APOB*, *TCN2*) transporters. The genes were calculated against glyceraldehyde-3-phosphate dehydrogenase (*GAPDH*). D: Western blotting (WB) analysis for FABP6, LYVE1, and CD31 in mucosa of colon from SBR control and SBR GW3965 piglets (each group, n = 4). Unpaired two-tailed Student’s *t* test with or without Welch’s correction analysis for (B, C, and E). Statistical significance: ns, not significant, ∗*P* < 0.05, ∗∗*P* < 0.01, ∗∗∗*P* < 0.001. FABP, fatty acid binding protein; LXR, liver X receptor; LYVE1, lymphatic vessel endothelial hyaluronan receptor 1; SBR, small-bowel resection.

We subsequently sought to elucidate whether LXR activation ameliorates the intestinal adaptation observed in SBR, particularly with respect to the transformation of colonic mucosa into an ileal phenotype. Remarkably, histopathological analysis revealed that treatment with GW3965 resulted in an increase in both quantity and complexity of villus-like structures within the colonic epithelial layer (Fig. 11A, B). IHC analysis further revealed a substantial elevation in the expression levels of ileal marker fatty acid-binding protein 2 (I-FABP) within the colonic mucosa following GW3965 treatment when compared to those observed in SBR controls (Fig. 11A, B). The qRT-PCR analysis demonstrated that GW3965 administration significantly upregulated the expression of genes associated with lipid (*ABCG5*, *FABP6*), amino acid (*SLC7A9*, *GLUL*), bile salt (*FGF19*, *SULT2A1*), and vitamin (*APOB*, *TCN2*) transporters—molecules typically absent or minimally expressed in colonic enterocytes (Fig. 11C). WB further corroborated these findings by revealing a substantial elevation in the expression levels of fatty acid binding protein 6 (FABP6), lymphatic vessel marker LYVE-1 and blood vessel marker CD31 within the colonic mucosa following GW3965 treatment when compared to those observed in SBR controls (Fig. 11D, E).

## Discussion

SBS is a clinical condition that arises from surgical resection, congenital defects, or disease-related loss of absorption, resulting in an inability to maintain nutrient balance when consuming a normal diet. The incidence of SBS is approximately 100-fold higher in preterm infants compared to term infants, primarily due to the increased occurrence of NEC in preterm infants as opposed to other congenital causes such as gastroschisis and intestinal atresia (21). Currently, therapeutic options for children with SBS remain limited. In this study, we demonstrated that activation of the LXR pathway promotes intestinal adaptation and regeneration in neonatal piglets following SBR, suggesting it may serve as a promising therapeutic strategy for children with SBS.

The condition of SBS arises from the surgical resection of the intestine, which initiates a process in the remaining intestinal tissue known as intestinal adaptation. This adaptation involves a coordinated increase in gut hormone secretion, mucosal growth, and secretory function. Many physiological processes associated with intestinal adaptation were initially described in early studies utilizing rat models (22), and subsequent research has extended these findings to murine models (23). Neonates diagnosed with SBS often rely on long-term PN therapy; however, many ultimately succumb to complications such as sepsis and liver disease. Research focused on neonatal SBS faces significant challenges due to ethical constraints surrounding the study of ill human neonates and the heterogeneous nature of this disease process. Outcomes for patients with SBS can vary considerably based on factors including residual intestinal anatomy, intestinal length, patient age, and exposure to nutritional therapies. From a comparative biology perspective, neonatal piglets represent an appropriate translational model for studying human neonates due to their similarities in gastrointestinal ontogeny, physiological maturity, and adaptive processes (24, 25, 26, 27). We have meticulously established SBR moles utilizing Bama piglets, approximately seven days of age, by conducting a 75% jejunoileal resection followed by a week of total parenteral nutrition (TPN). This approach encapsulates the full spectrum of pathologies observed in human infants, thereby serving as an exemplary translational animal model.

The nuclear receptors, known as LXRs, have been recognized for their pivotal role in the complex transcriptional regulation of lipid and cholesterol metabolism (11, 12). LXR activation has demonstrated remarkable efficacy in activating the LXR pathway, thereby enhancing the reparative processes within the intestine following injury in murine models (14). LXR agonists have now been shown to ameliorate pathology in multiple preclinical rodent models of inflammatory disease (28). Most studies to date have used the first-generation dual LXRα/β agonists TO901317 and GW3965, primarily demonstrating their efficacy in models of inflammatory bowel disease such as dextran sulfate sodium-induced colitis (29). However, the therapeutic potential of LXR agonism remains entirely unexplored in the context of SBS.

In this study, we investigate LXR activation using synthetic agonists such as GW3965 in a model involving neonatal SBR piglets. Our observations reveal that administration of GW3965 not only induced robust LXR activation in these SBR piglets, but also facilitated intestinal adaptation by ameliorating inflammation associated with intestinal injuries, fortifying the intestinal barrier, and promoting regenerative processes within the gut. The integrity of the intestinal barrier represents a multifaceted defense and regulatory system composed of an array of functionally intricate cells and biomolecules. This system encompasses mechanical, chemical, immune, and biological barriers that collectively safeguard against bacterial infiltration and endotoxin exposure while preserving the delicate ecological balance of the intestinal environment (30). Disruption of the intestinal barrier attributed to SBS leads to bacterial translocation, endotoxin absorption, and intestinal inflammation. These complications significantly delay the recovery of intestinal function (31, 32, 33). Our findings indicate that LXR activation not only enhances mucin production from goblet cells but also improves tight junction integrity by increasing the expression of proteins such as ZO-1, Claudin-1, and Occludin. Furthermore, we observed a reduction in serum LPS levels in GW3965-treated piglets, which subsequently attenuated TLR4-NFκB inflammatory signaling. In mouse models subjected to irradiation and dextran sodium sulfate-induced damage in both the small intestine (SI) and colon, Srustidhar Das *et al.* reported that LXR pathway activation reciprocally regulates intestinal regeneration and tumorigenesis (14), in which They demonstrated that LXR activation in intestinal epithelial cells induces AREG, thereby enhancing regenerative responses. In our present study, treatment with GW3965 resulted in increased growth of the intestinal mucosa while inhibiting epithelial apoptosis. Consistently, we also noted an increase in EGF ligand AREG expression along with another ligand EREG following GW3965 treatment. In the realm of human organoids, we propose that GW3965 facilitating crypt growth is associated with the induction of AREG and EREG, suggesting a potential mechanism for the enhanced proliferation. Future studies using EGFR pathway inhibitors are necessary to establish a causal link Organoids derived from the ileum, when introduced into the colonic epithelium of rats, successfully restored absorptive function and circumvented intestinal failure, (34) but the clinical potential of this strategy is constrained by factors such as cost, scalability, and intricate surgical procedures. Remarkably, our findings indicated that LXR activation prompted the formation of villus-like structures within the colon while simultaneously enhancing the expression of small intestine-specific genes responsible for transporting lipids, amino acids, bile salts, and vitamins—elements typically absent or present at minimal levels in colonic tissue. Furthermore, LXR activation also stimulated lymphatic and blood vessel development within the colons. However, functional validation through assays of bile acid or vitamin B12 absorption is required to confirm that this molecular shift translates into a clinically significant adaptive function. Cholestanetriol 26-monooxygenase (CYP27A1) has been reported acted as upstream of LXR, which produced LXR-ligand (14). The cyp27a1 gene is an enzyme that transforms the cholesterol into primary bile acids and/or a series of cholesterol derivatives, which has been demonstrated to associated with relative abundance of Phaeobacter. We thus speculated that microbiota may participates in intestinal homeostasis partly affected LXR pathway.

This study has several important limitations that should be considered when interpreting the results. Firstly, the small sample size in the GW3965-treated group (n = 4) limits the statistical power for some endpoints and the assessment of inter-piglet variability. These findings, while promising, require confirmation in a larger, fully-powered cohort. Secondly, and most critically, our experimental model utilized exclusive postoperative TPN without enteral feeding and lacked a sham-operated control group (transection and reanastomosis without resection). Consequently, the phenotypic changes we observed represent a composite of the intestinal response to resection, the pathological effects of TPN-induced gut atrophy, and the systemic inflammatory response to major surgery. While our data robustly show that LXR agonist treatment modifies this severe clinical phenotype, we cannot definitively isolate its specific effect on the process of true intestinal adaptation from its potential modulation of surgical stress or TPN toxicity. Future studies incorporating enteral feeding and sham controls are essential to dissect these mechanisms. Furthermore, the anatomical configuration of our SBS model, which preserved a longer ileal segment, models a specific clinical scenario (e.g., proximal jejunal resection) but differs from the more common situation of a jejunal remnant. The therapeutic response may vary based on the primary site of the remaining bowel. Finally, while we demonstrate a beneficial effect of LXR activation, the precise molecular mechanisms within the intestinal mucosa remain to be fully elucidated. Preclinical studies are also necessary to thoroughly validate the safety and potential adverse effects of chronic LXR agonist administration before clinical translation can be considered.

The successful establishment of the neonatal piglet SBS model in this study provides a robust platform for subsequent investigation. Building upon these initial promising findings, several critical research directions are warranted. First, the results of this study must be validated in a larger cohort to ensure statistical robustness, account for inter-animal variability, and solidify the observed therapeutic effect of LXR agonism. Second, a comprehensive preclinical safety and toxicology profile of the LXR agonist is essential. Future studies will need to systematically evaluate potential adverse effects, particularly concerning lipid metabolism and hepatic steatosis, which are known class effects of LXR agonists, to assess its clinical translatability. Third, a direct, head-to-head comparison with the current standard of care is crucial. The most informative next step would be to evaluate the efficacy of the LXR agonist against and in combination with GLP-2 analogs (e.g., teduglutide), which are already approved for the treatment of SBS in children and adults. Such a study would determine the relative potency and potential synergistic effects of these distinct therapeutic pathways. Finally, the underlying mechanisms require deeper elucidation. Future work should employ transcriptomic and proteomic approaches to identify the specific downstream targets of LXR signaling in the adapting intestine and investigate its interplay with other critical pathways, including those influenced by the gut microbiome.

In summary, we successfully established and characterized a neonatal piglet model of SBS that recapitulates key pathophysiological features observed in pediatric patients. Using this model, we demonstrated that treatment with the LXR agonist GW3965 significantly ameliorates intestinal failure by enhancing adaptive responses, improving intestinal barrier function, suppressing systemic inflammation, and promoting mucosal regeneration. These promising results suggest that pharmacological activation of the LXR pathway holds significant potential as a novel therapeutic strategy for intestinal rehabilitation in SBS. Further investigation is warranted to validate these findings and elucidate the precise mechanisms of action.

## Data availability

The data generated or analyzed during this study are available from the corresponding author upon reasonable request.

## Supplemental data

This article contains supplemental data.

## Conflicts of interest

The authors declare that they have no conflicts of interest with the contents of this article.
