# Supplementary material for: Liver X receptor agonists enhance intestinal repair in neonatal piglets with massive bowel resection
Source: J Lipid Res. 2026 May 28;67(7):101071. doi: 10.1016/j.jlr.2026.101071 (PMC13315804; doi:10.1016/j.jlr.2026.101071)
Supplement: Supporting information [file mmc1.pdf]

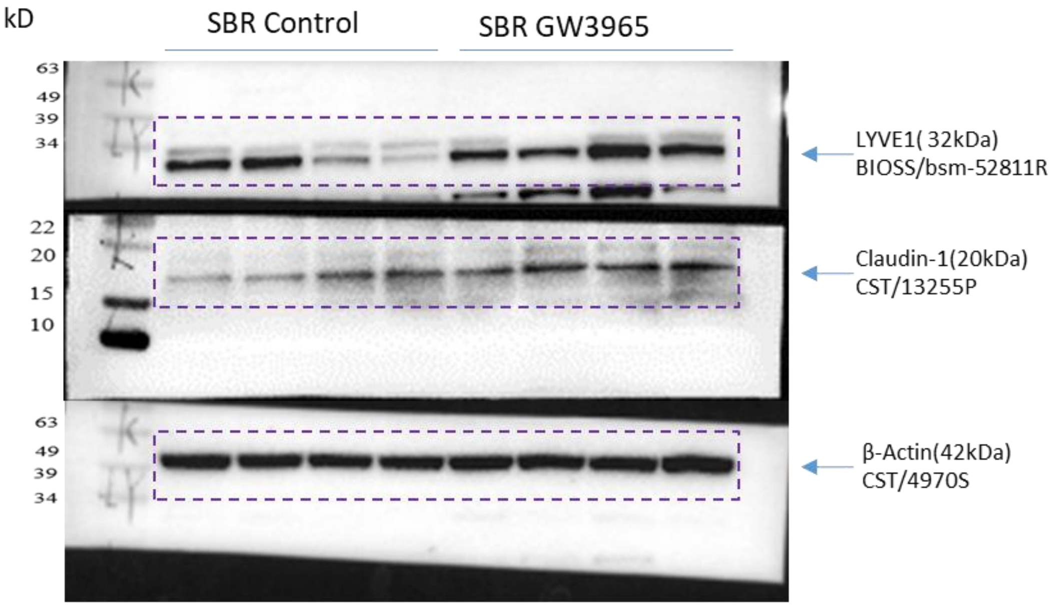
kD
SBR Control
SBR GW3965
63
49
39
34
22
20
15
10
63
49
39
34
LYVE1(32kDa)
BIOSS/bsm-52811R
Claudin-1(20kDa)
CST/13255P
β-Actin(42kDa)
CST/4970S

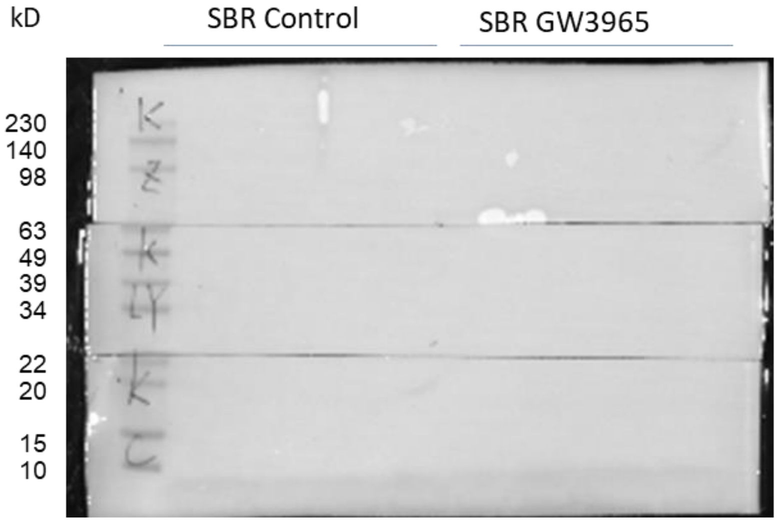
kD
SBR Control
SBR GW3965
230
140
98
63
49
39
34
22
20
15
10

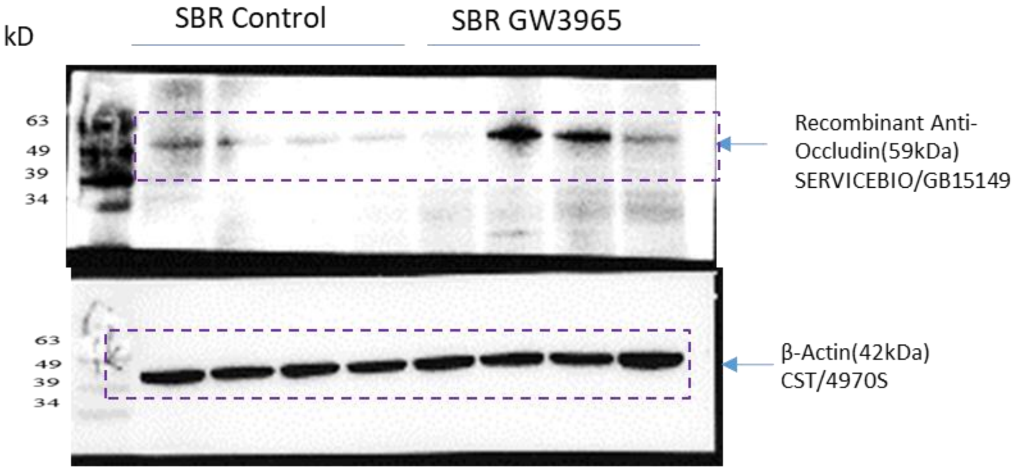
SBR Control
SBR GW3965
kD
63
49
39
34
Recombinant Anti-Occludin(59kDa)
SERVICEBIO/GB15149
63
49
39
34
β-Actin(42kDa)
CST/4970S

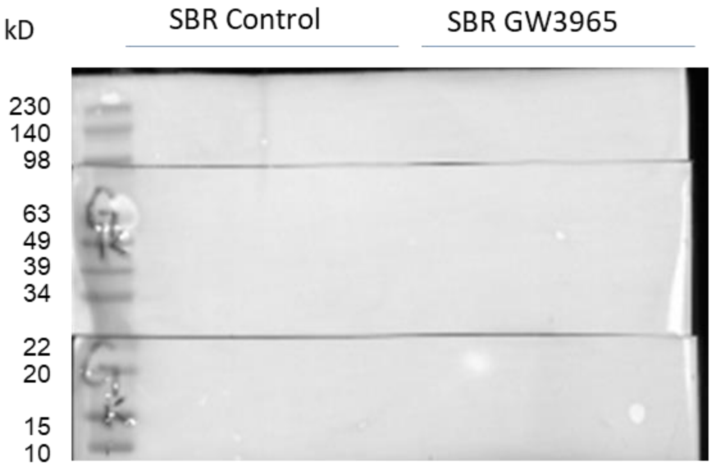
kD
SBR Control
SBR GW3965
230
140
98
63
49
39
34
22
20
15
10

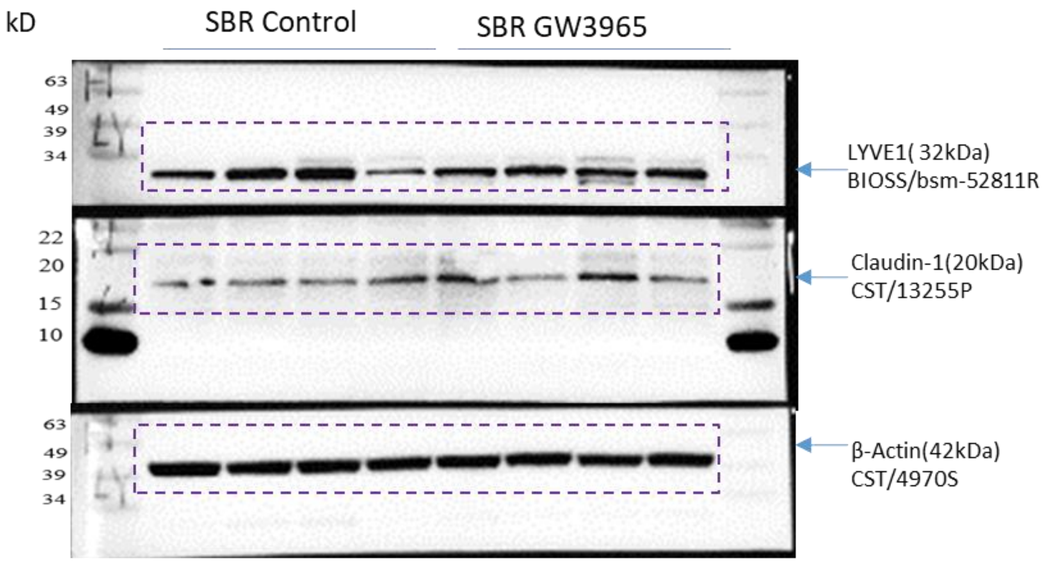
kD
SBR Control
SBR GW3965
63
49
39
34
22
20
15
10
63
49
39
34
LYVE1(32kDa)
BIOSS/bsm-52811R
Claudin-1(20kDa)
CST/13255P
β-Actin(42kDa)
CST/4970S

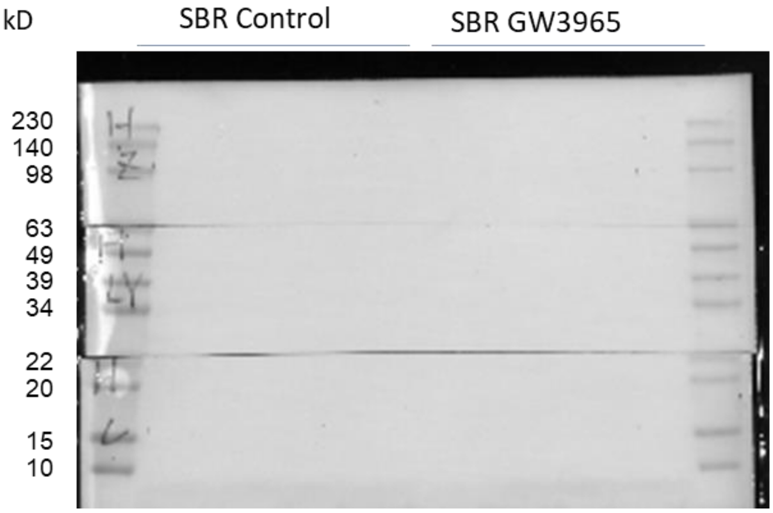
kD
SBR Control
SBR GW3965
230
140
98
63
49
39
34
22
20
15
10

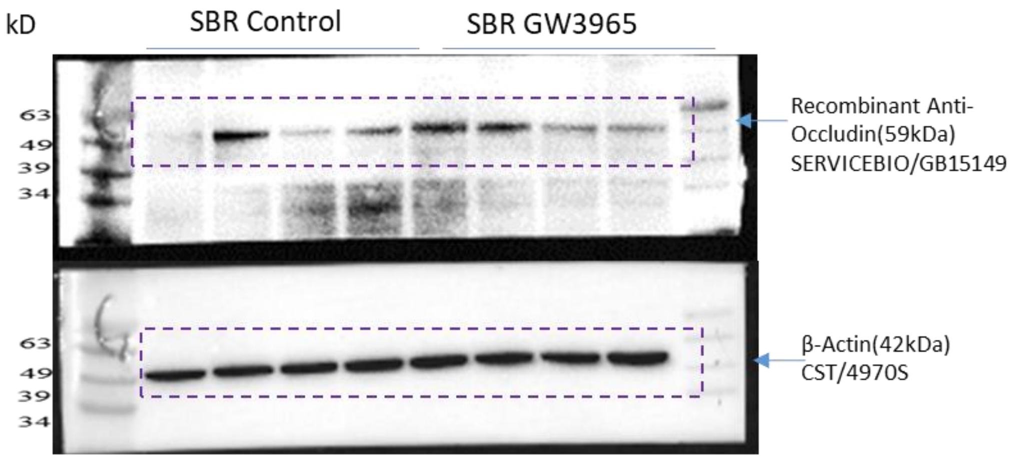
kD
SBR Control
SBR GW3965
63
49
39
34
Recombinant Anti-Occludin(59kDa)
SERVICEBIO/GB15149
63
49
39
34
β-Actin(42kDa)
CST/4970S

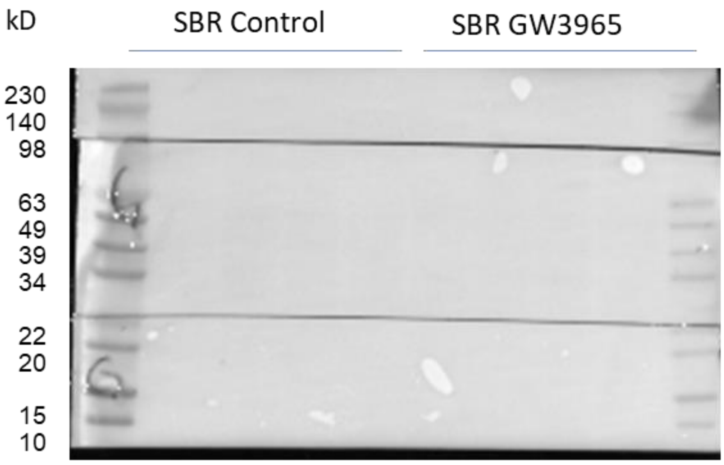
kD
SBR Control
SBR GW3965
230
140
98
63
49
39
34
22
20
15
10

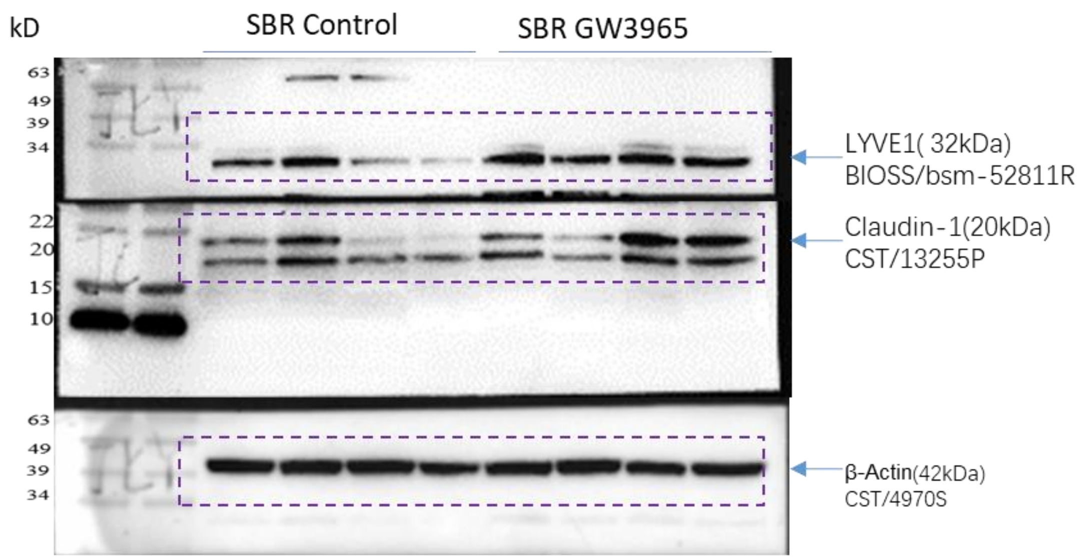

kD
SBR Control
SBR GW3965
63
49
39
34
22
20
15
10
63
49
39
34
LYVE1(32kDa)
BIOSS/bsm-52811R
Claudin-1(20kDa)
CST/13255P
β-Actin(42kDa)
CST/4970S

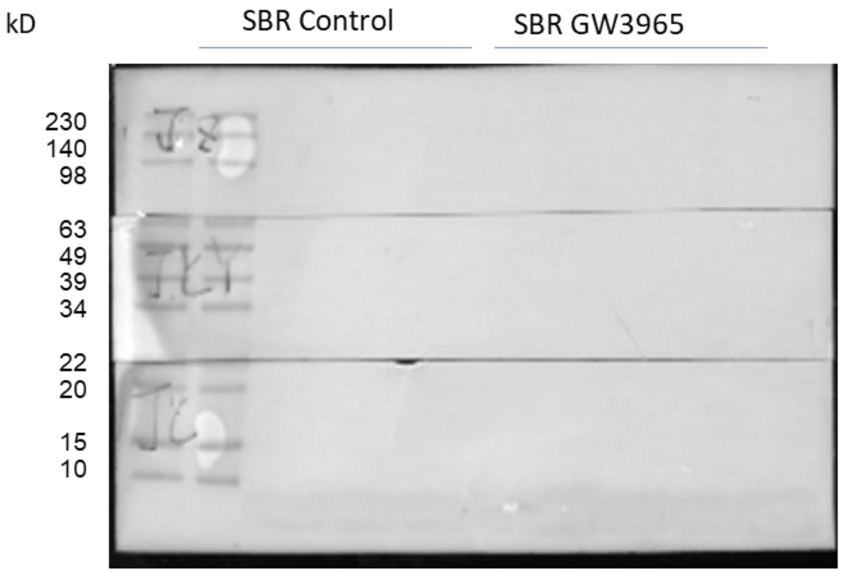

kD
SBR Control
SBR GW3965
230
140
98
63
49
39
34
22
20
15
10

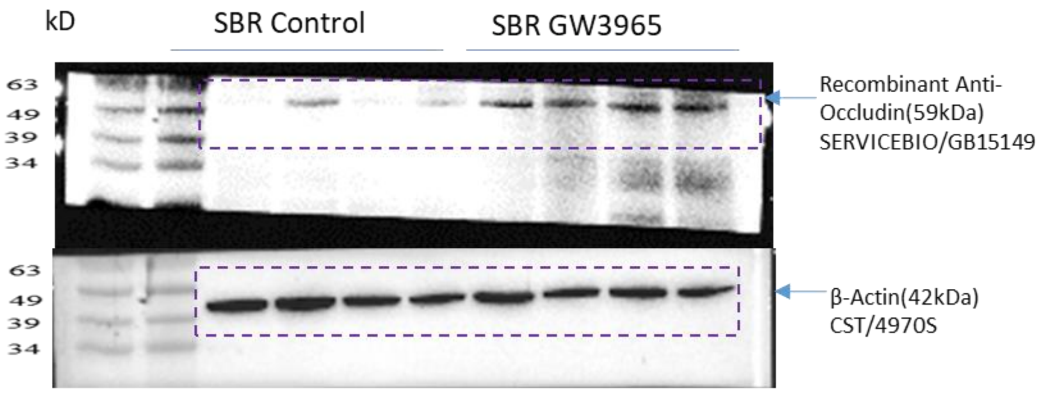
kD
SBR Control
SBR GW3965
63
49
39
34
Recombinant Anti-
Occludin(59kDa)
SERVICEBIO/GB15149
63
49
39
34
β-Actin(42kDa)
CST/4970S

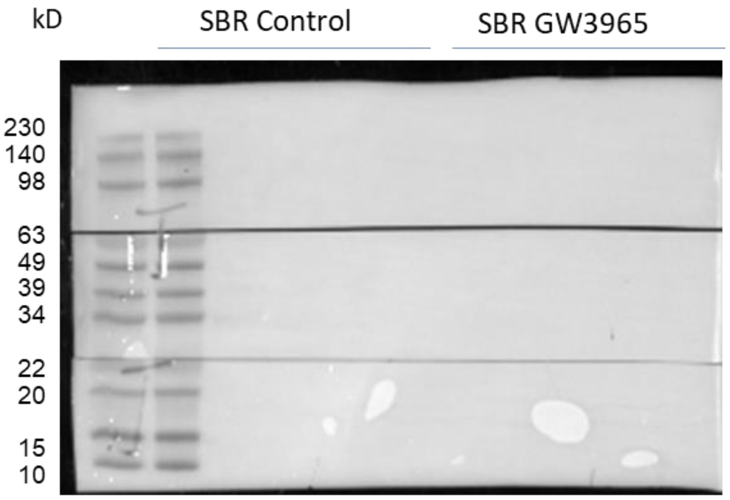
kD
SBR Control
SBR GW3965
230
140
98
63
49
39
34
22
20
15
10

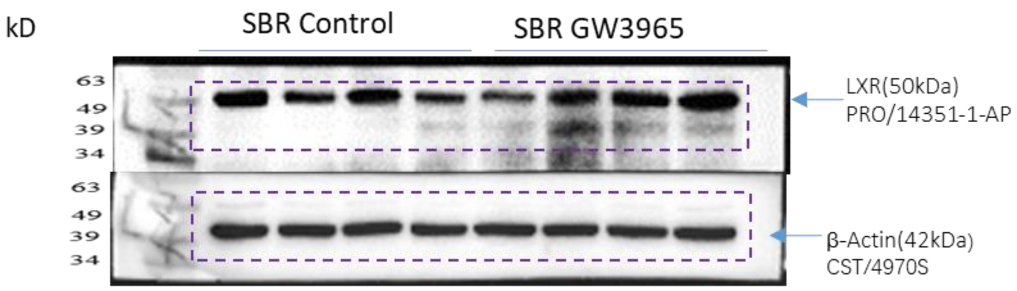
kD
SBR Control
SBR GW3965
63
49
39
34
63
49
39
34
LXR(50kDa)
PRO/14351-1-AP
β-Actin(42kDa)
CST/4970S

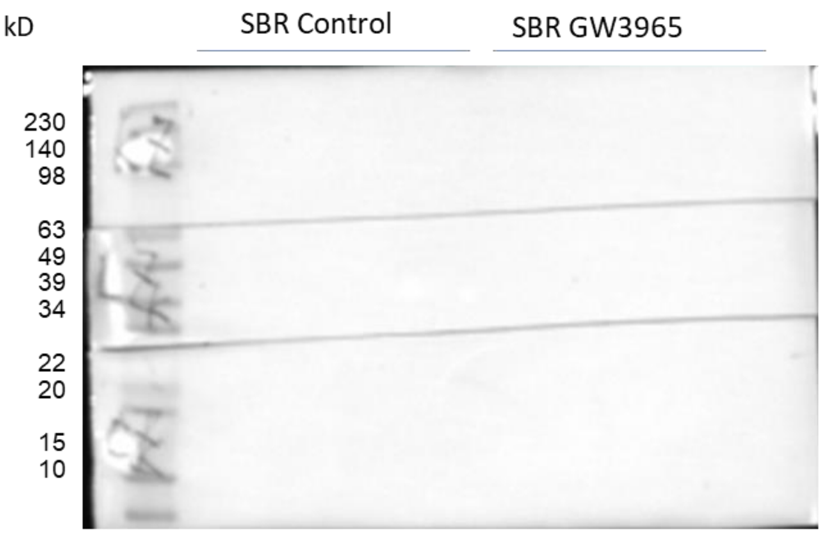
kD
SBR Control
SBR GW3965
230
140
98
63
49
39
34
22
20
15
10

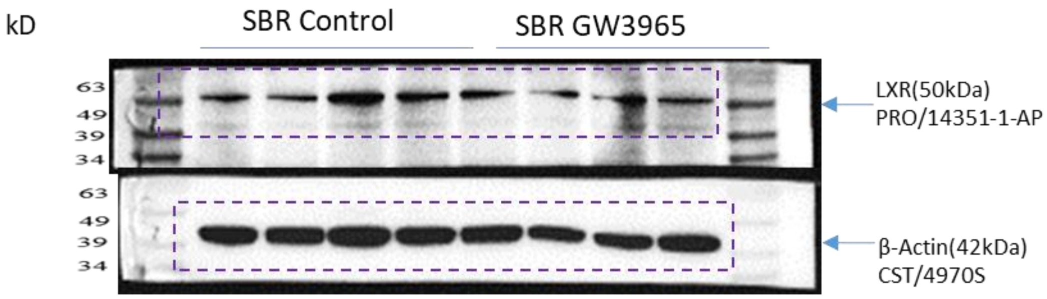
kD
SBR Control
SBR GW3965
63
49
39
34
63
49
39
34
LXR(50kDa)
PRO/14351-1-AP
β-Actin(42kDa)
CST/4970S

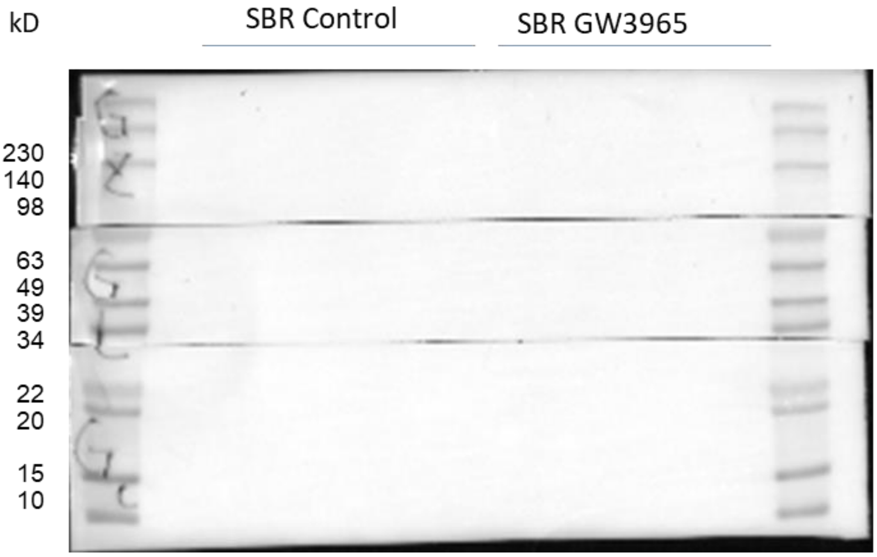
kD
SBR Control
SBR GW3965
230
140
98
63
49
39
34
22
20
15
10

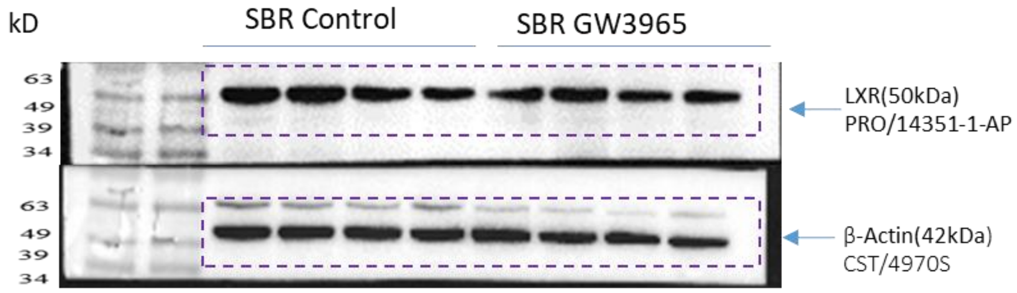
kD
SBR Control
SBR GW3965
63
49
39
34
LXR(50kDa)
PRO/14351-1-AP
63
49
39
34
β-Actin(42kDa)
CST/4970S

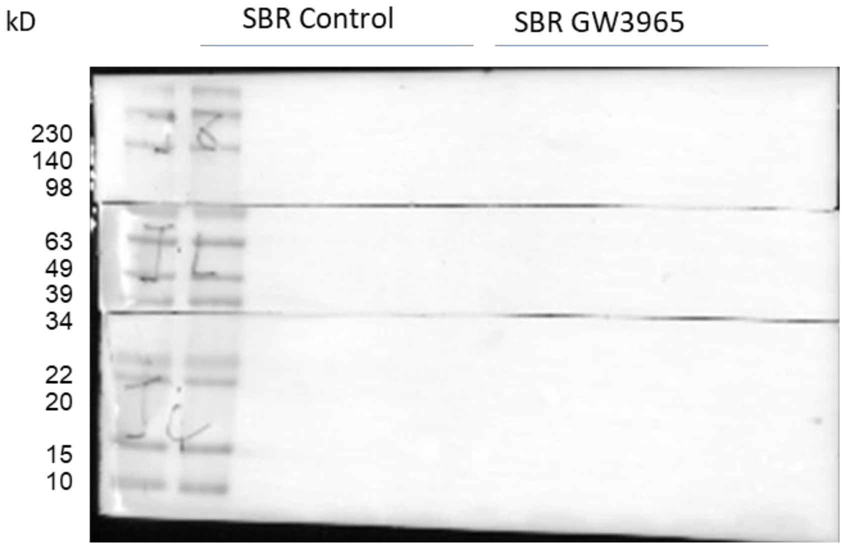
kD
SBR Control
SBR GW3965
230
140
98
63
49
39
34
22
20
15
10

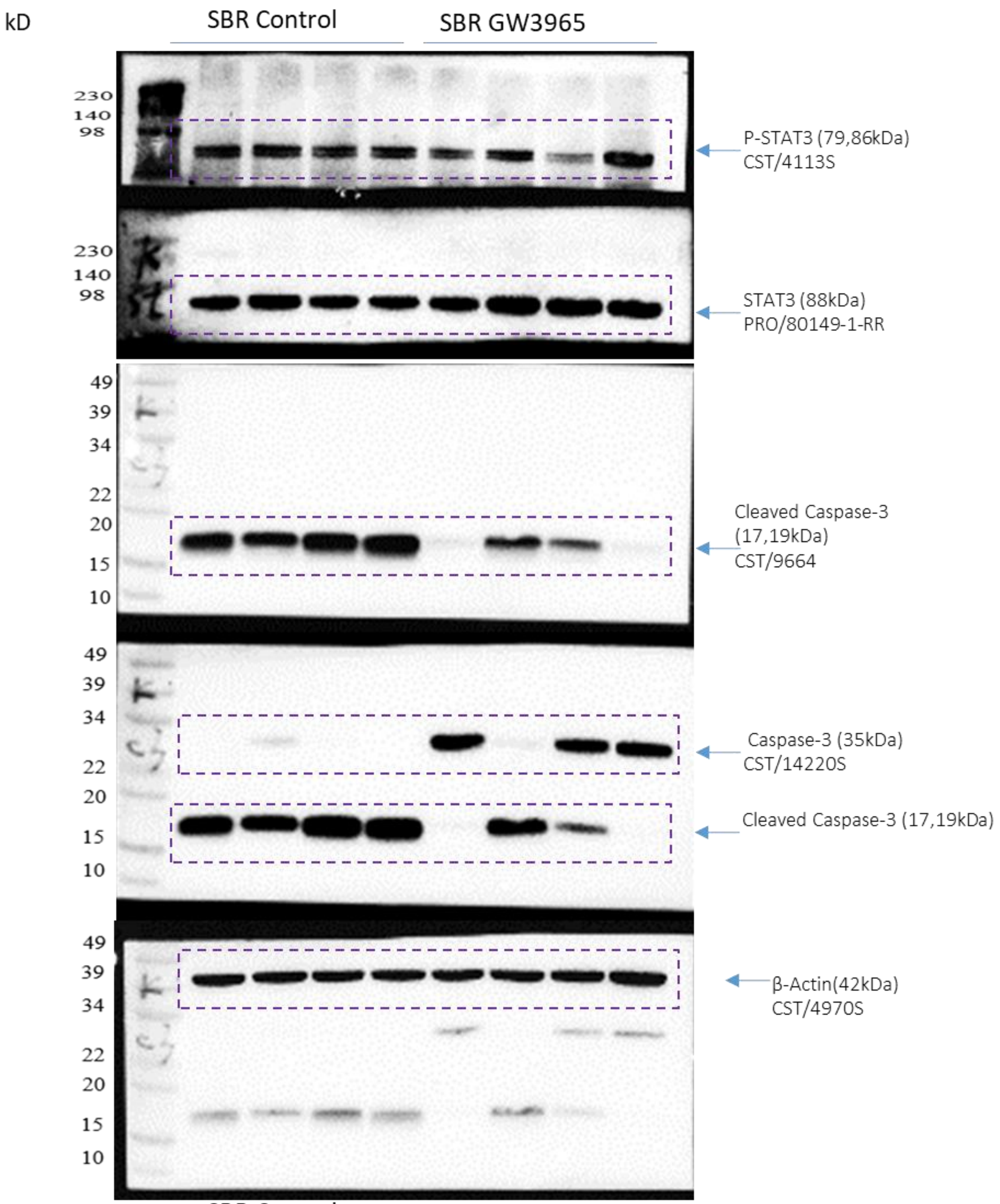
kD
SBR Control
SBR GW3965
230
140
98
P-STAT3 (79,86kDa)
CST/4113S
230
140
98
STAT3 (88kDa)
PRO/80149-1-RR
49
39
34
22
20
15
10
Cleaved Caspase-3
(17,19kDa)
CST/9664
49
39
34
22
20
15
10
Caspase-3 (35kDa)
CST/14220S
Cleaved Caspase-3 (17,19kDa)
49
39
34
22
20
15
10
β-Actin(42kDa)
CST/4970S

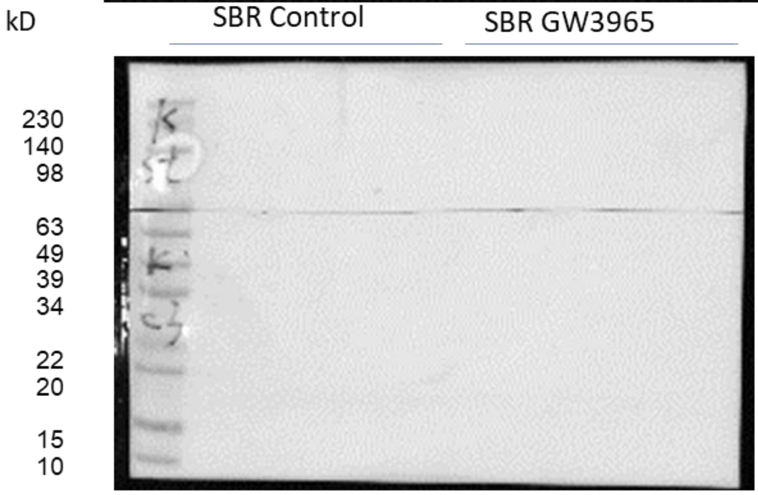
kD
SBR Control
SBR GW3965
230
140
98
63
49
39
34
22
20
15
10

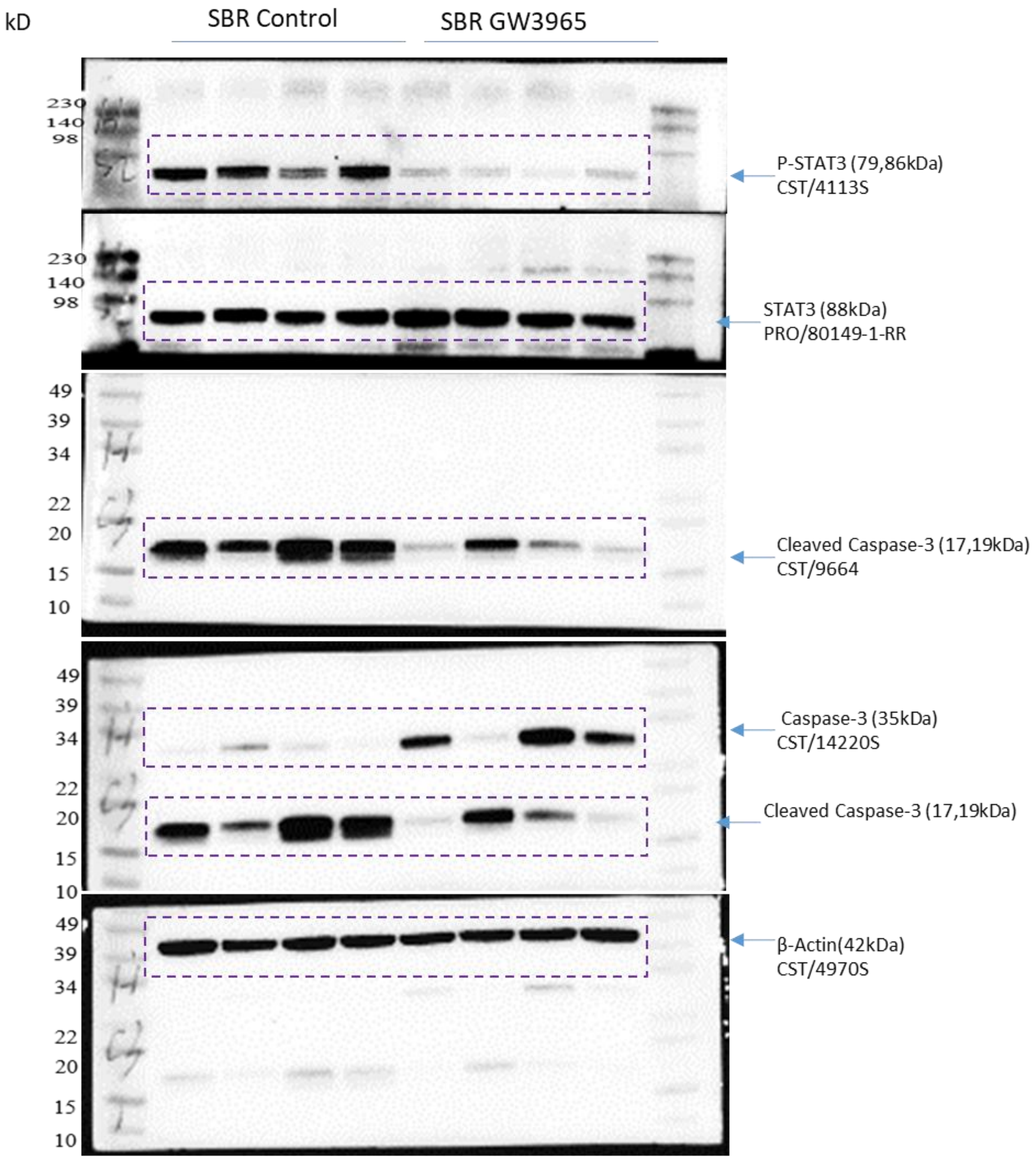
kD
SBR Control
SBR GW3965
P-STAT3 (79,86kDa)
CST/4113S
STAT3 (88kDa)
PRO/80149-1-RR
Cleaved Caspase-3 (17,19kDa)
CST/9664
Caspase-3 (35kDa)
CST/14220S
Cleaved Caspase-3 (17,19kDa)
β-Actin(42kDa)
CST/4970S
230
140
98
49
39
34
22
20
15
10

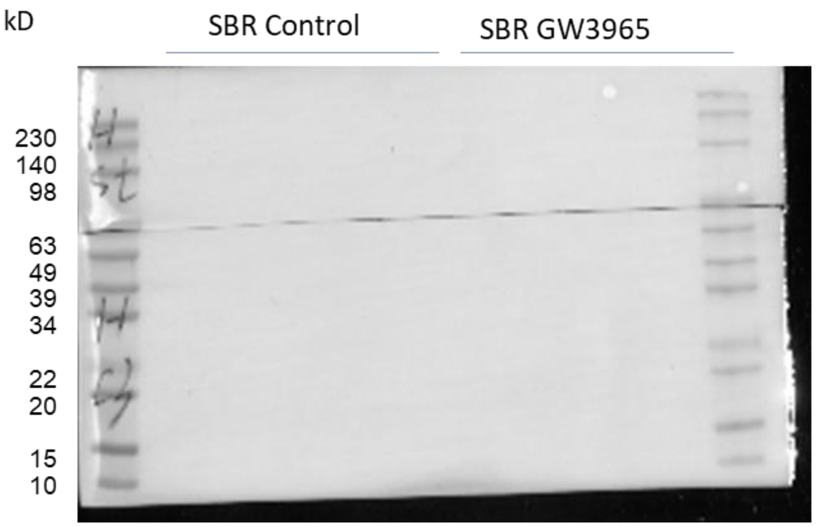
kD
SBR Control
SBR GW3965
230
140
98
63
49
39
34
22
20
15
10

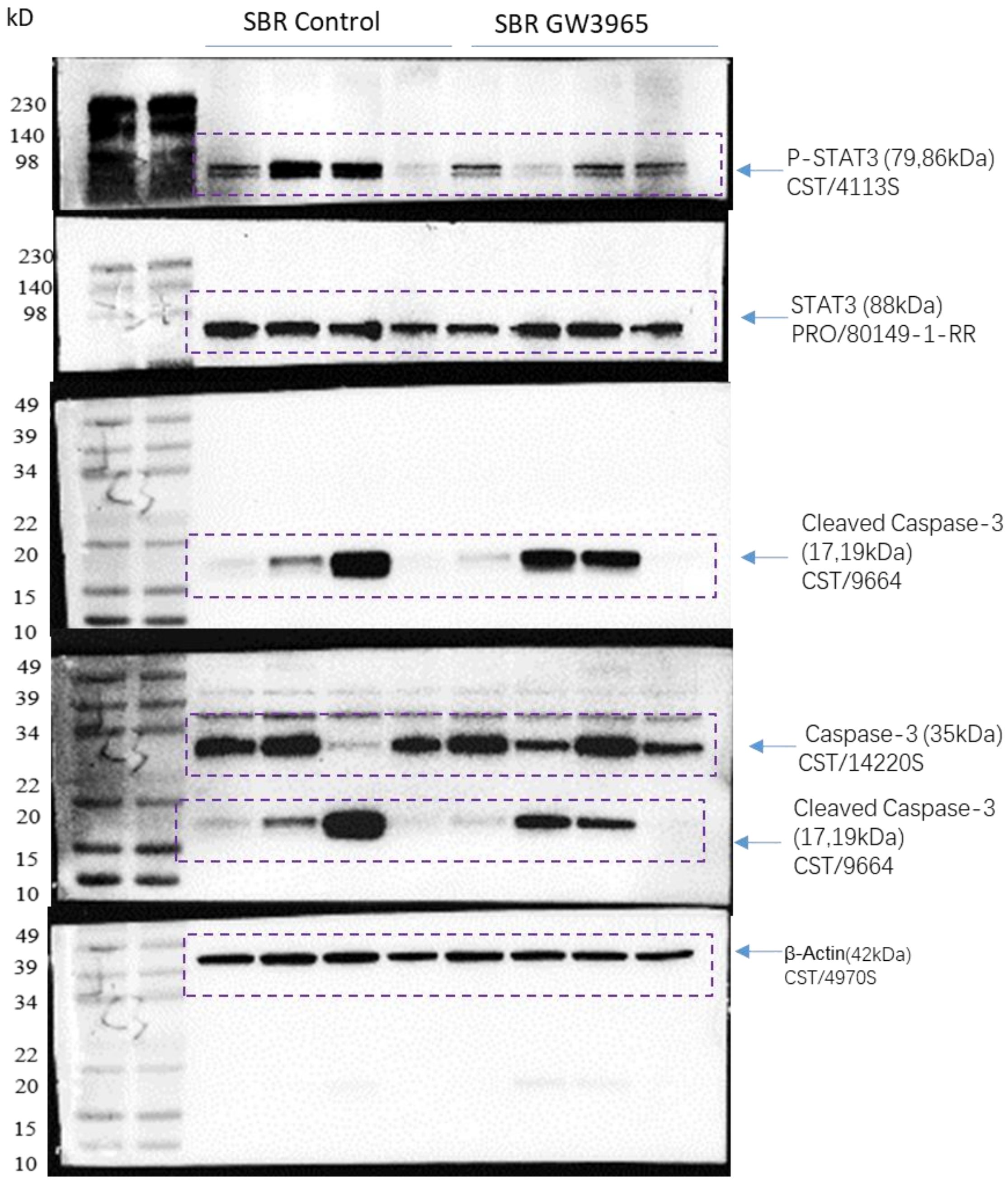
kD
SBR Control
SBR GW3965
230
140
98
P-STAT3 (79,86kDa)
CST/4113S
230
140
98
STAT3 (88kDa)
PRO/80149-1-RR
49
39
34
22
20
15
10
Cleaved Caspase-3
(17,19kDa)
CST/9664
49
39
34
22
20
15
10
Caspase-3 (35kDa)
CST/14220S
Cleaved Caspase-3
(17,19kDa)
CST/9664
49
39
34
22
20
15
10
β-Actin(42kDa)
CST/4970S

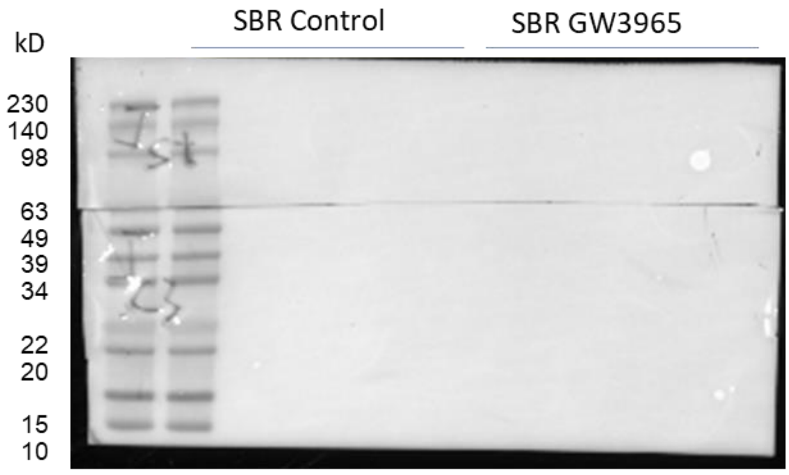
kD
SBR Control
SBR GW3965
230
140
98
63
49
39
34
22
20
15
10

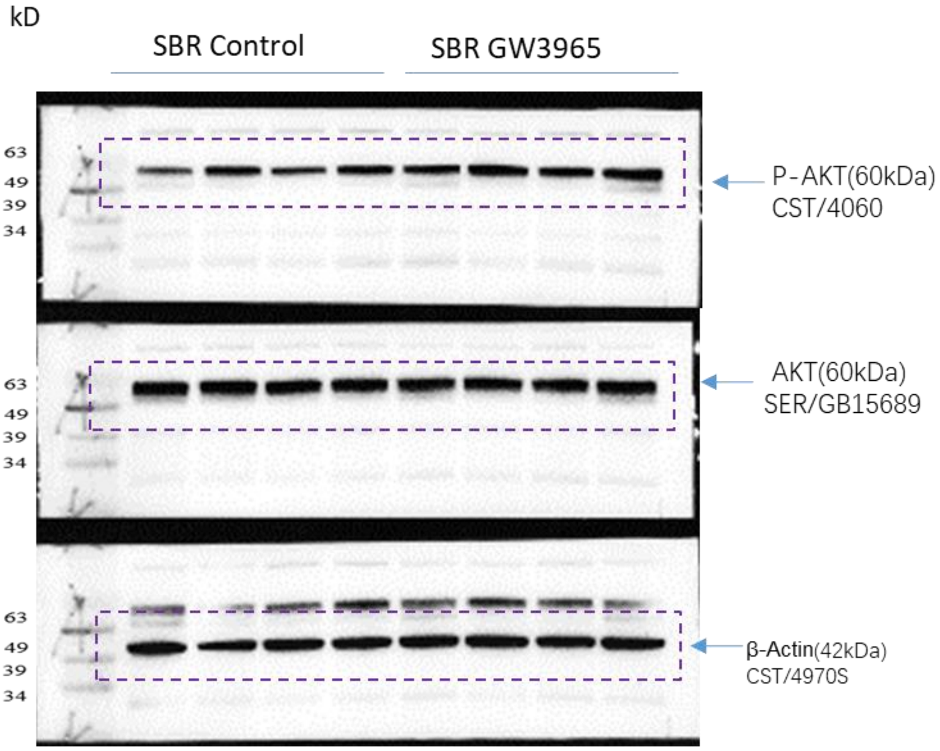
kD
SBR Control
SBR GW3965
63
49
39
34
P-AKT(60kDa)
CST/4060
AKT(60kDa)
SER/GB15689
β-Actin(42kDa)
CST/4970S

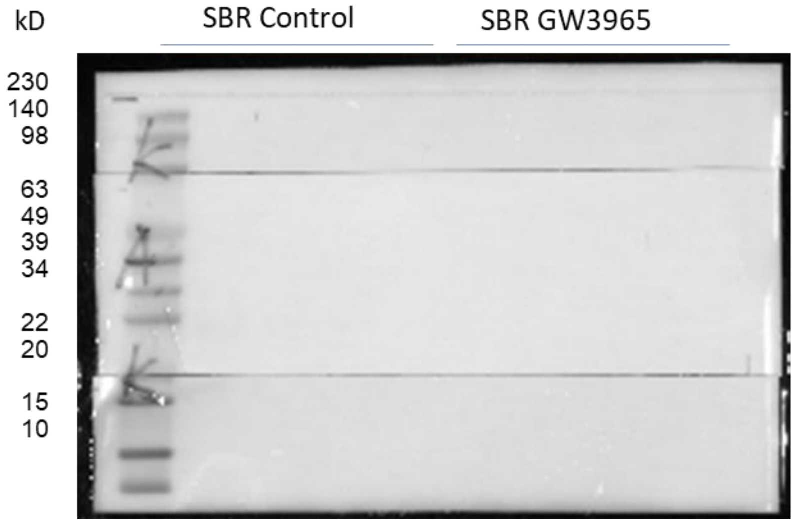
kD
SBR Control
SBR GW3965
230
140
98
63
49
39
34
22
20
15
10

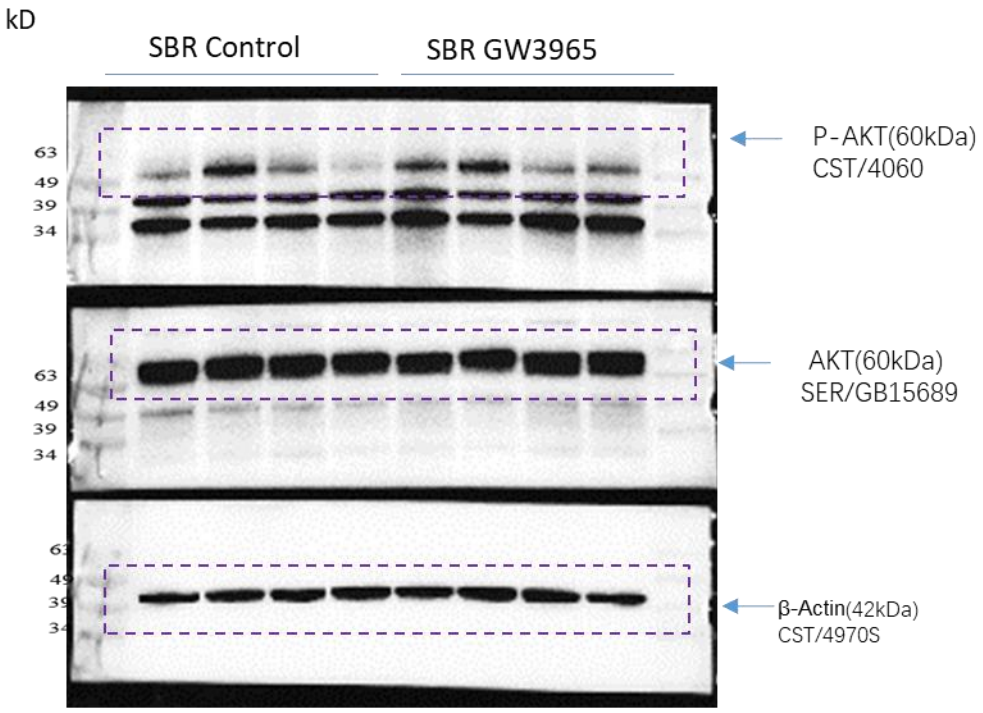
kD
SBR Control
SBR GW3965
63
49
39
34
P-AKT(60kDa)
CST/4060
AKT(60kDa)
SER/GB15689
β-Actin(42kDa)
CST/4970S

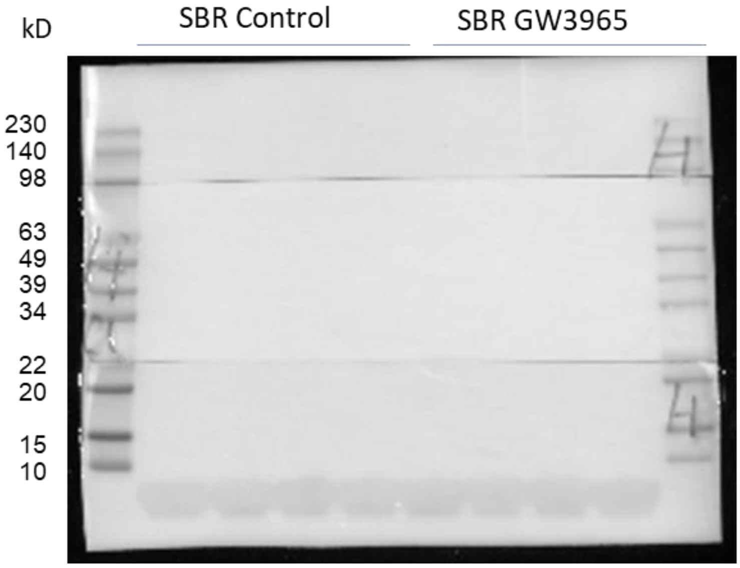
kD
SBR Control
SBR GW3965
230
140
98
63
49
39
34
22
20
15
10

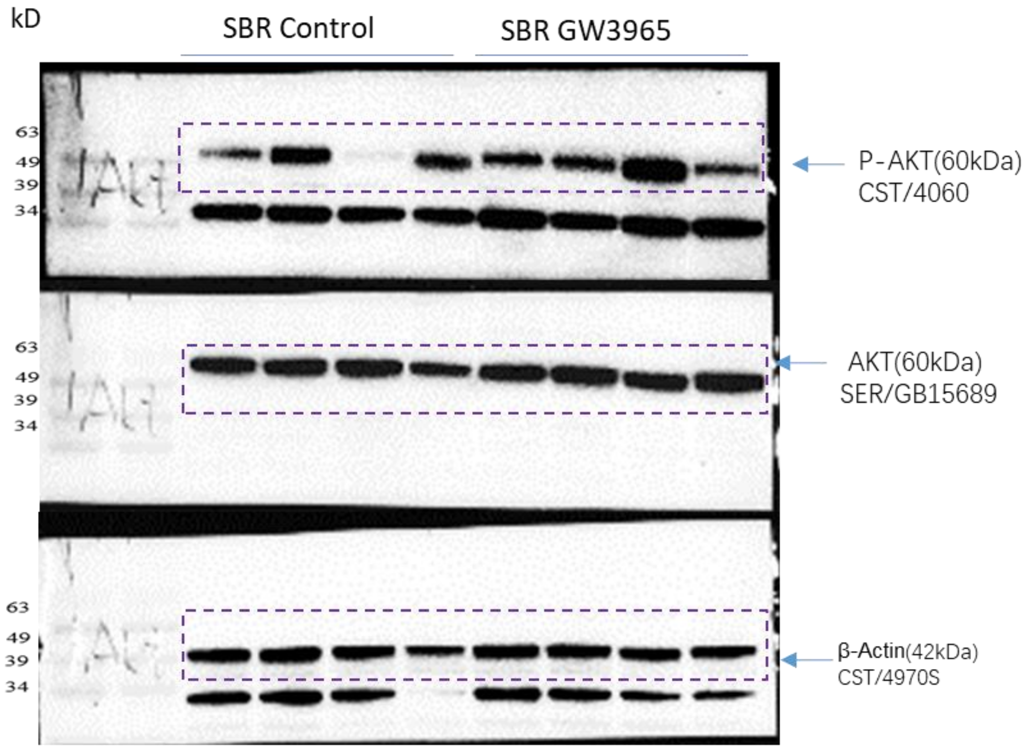
kD
SBR Control
SBR GW3965
63
49
39
34
P-AKT(60kDa)
CST/4060
AKT(60kDa)
SER/GB15689
β-Actin(42kDa)
CST/4970S

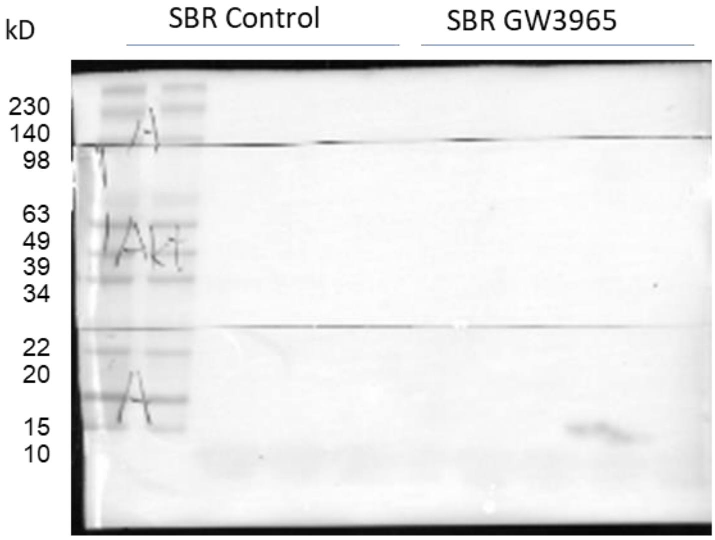
kD
SBR Control
SBR GW3965
230
140
98
63
49
39
34
22
20
15
10

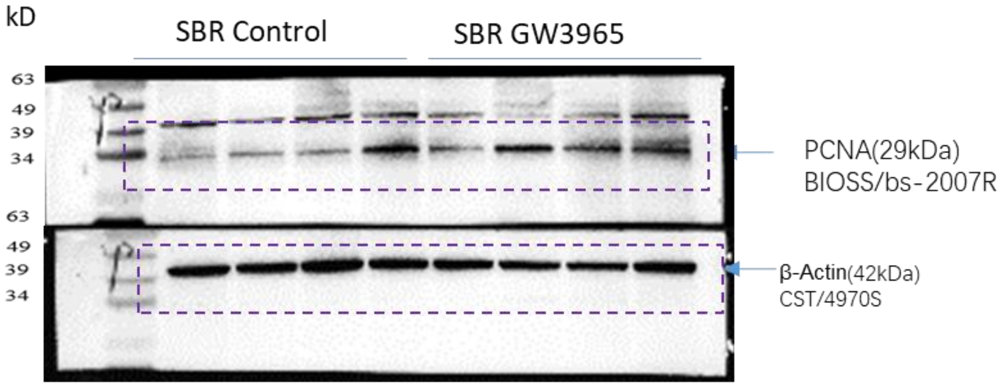
kD
SBR Control
SBR GW3965
63
49
39
34
63
49
39
34
PCNA(29kDa)
BIOSS/bs-2007R
β-Actin(42kDa)
CST/4970S

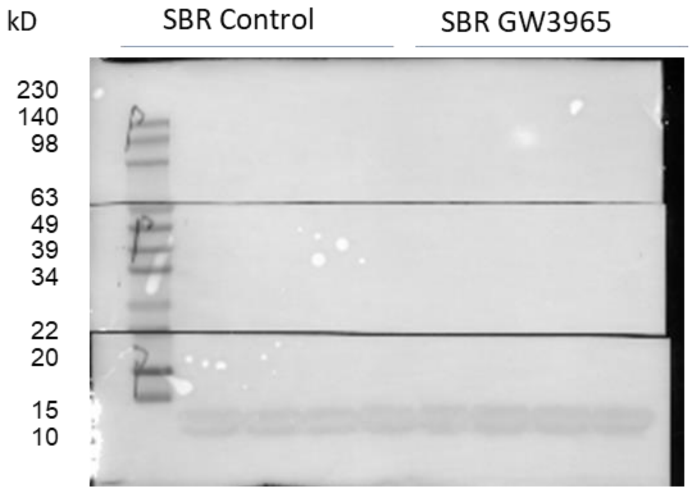
kD
SBR Control
SBR GW3965
230
140
98
63
49
39
34
22
20
15
10

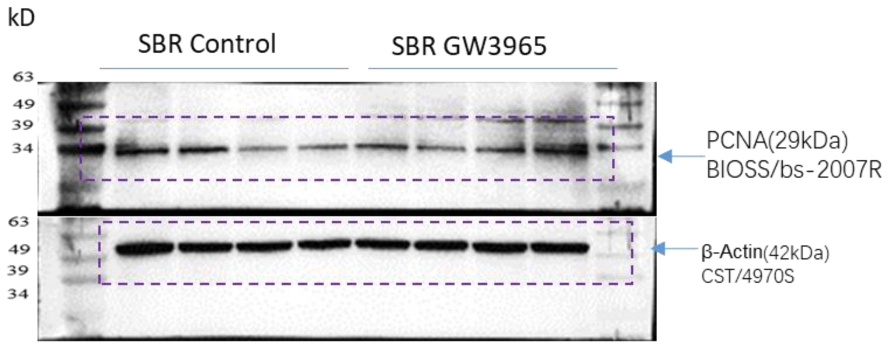
kD
SBR Control
SBR GW3965
63
49
39
34
63
49
39
34
PCNA(29kDa)
BIOSS/bs-2007R
β-Actin(42kDa)
CST/4970S

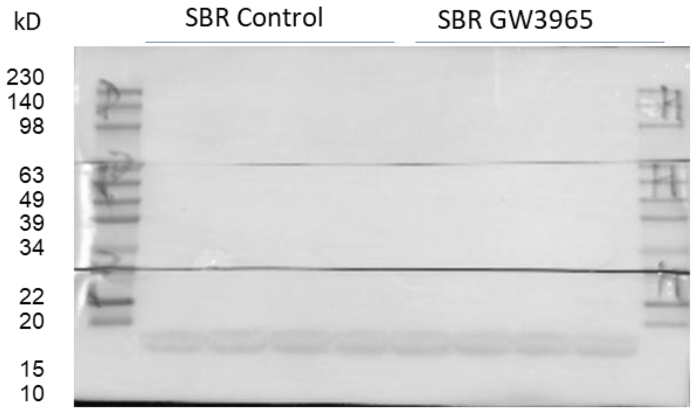
kD
SBR Control
SBR GW3965
230
140
98
63
49
39
34
22
20
15
10

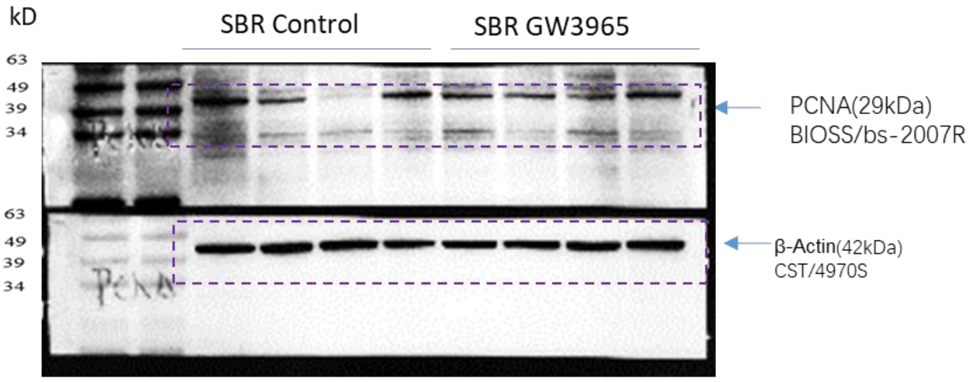
kD
SBR Control
SBR GW3965
63
49
39
34
63
49
39
34
PCNA(29kDa)
BIOSS/bs-2007R
β-Actin(42kDa)
CST/4970S

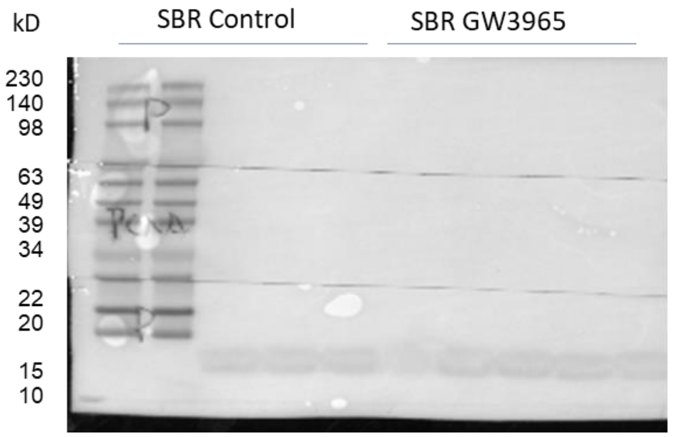
kD
SBR Control
SBR GW3965
230
140
98
63
49
39
34
22
20
15
10

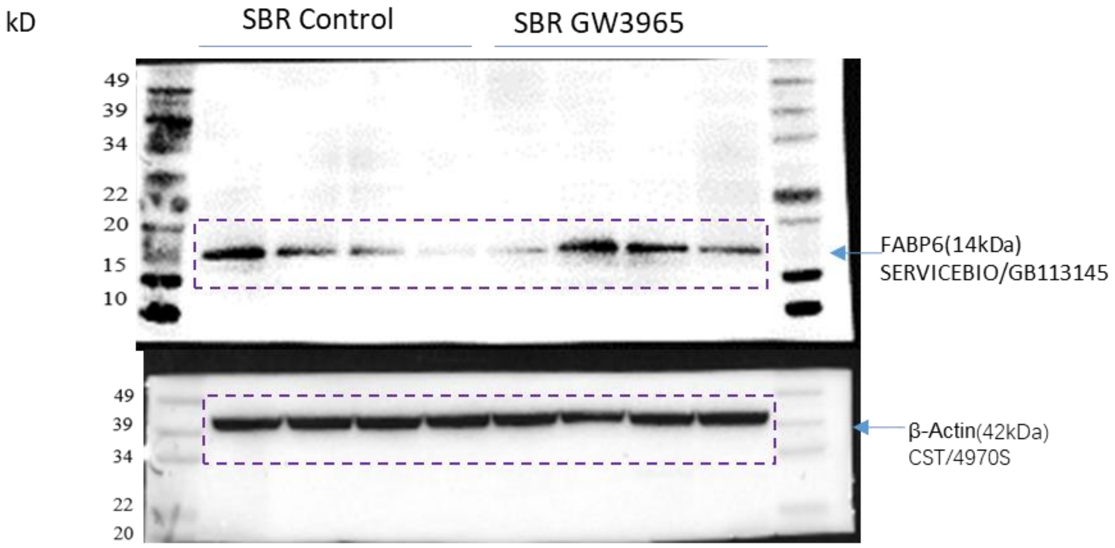
kD
SBR Control
SBR GW3965
49
39
34
22
20
15
10
FABP6(14kDa)
SERVICEBIO/GB113145
49
39
34
22
20
β-Actin(42kDa)
CST/4970S

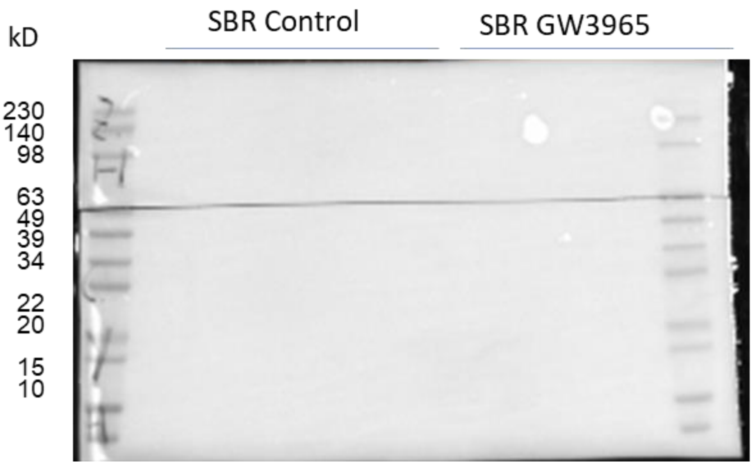
kD
SBR Control
SBR GW3965
230
140
98
63
49
39
34
22
20
15
10

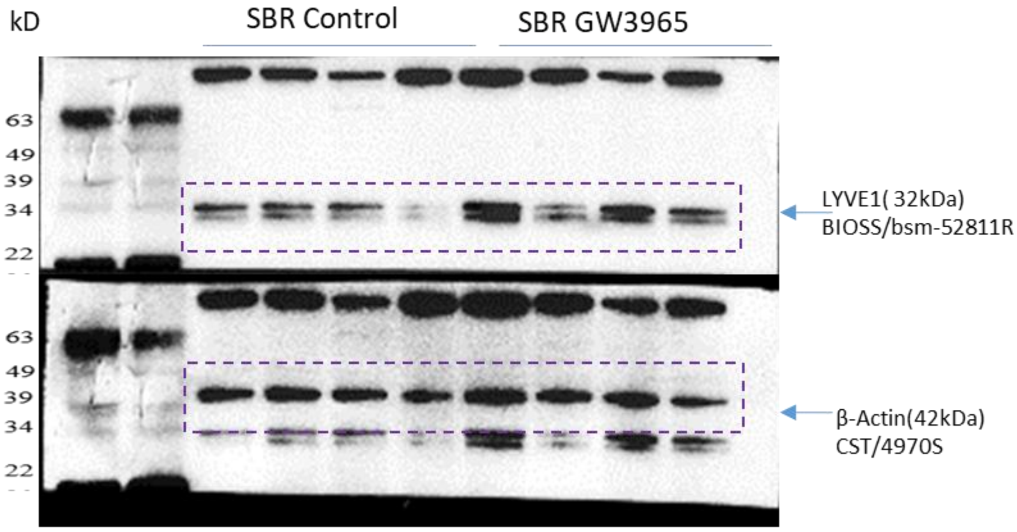
kD
SBR Control
SBR GW3965
63
49
39
34
22
LYVE1(32kDa)
BIOSS/bsm-52811R
β-Actin(42kDa)
CST/4970S

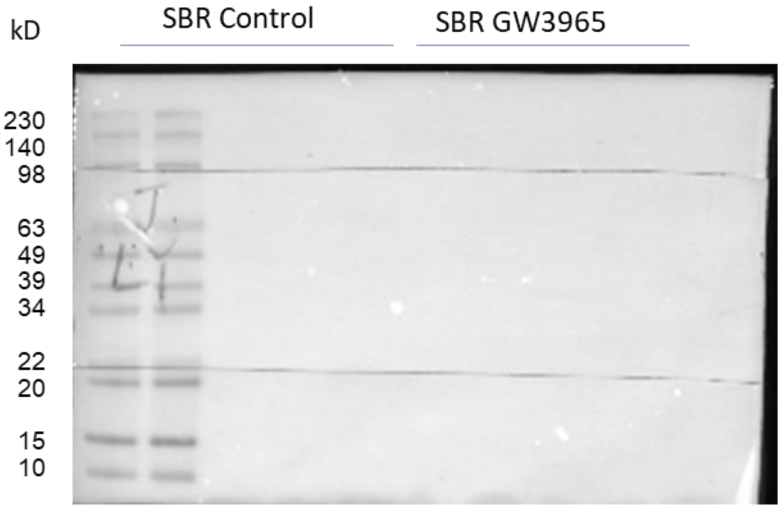
kD
SBR Control
SBR GW3965
230
140
98
63
49
39
34
22
20
15
10

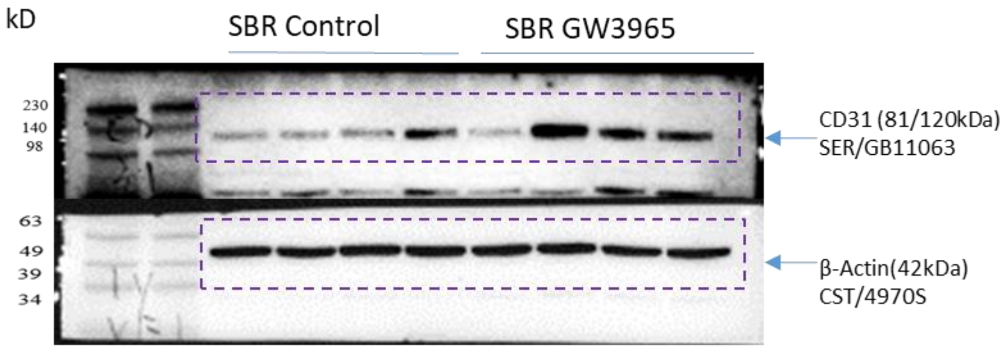
kD
SBR Control
SBR GW3965
230
140
98
63
49
39
34
CD31 (81/120kDa)
SER/GB11063
β-Actin(42kDa)
CST/4970S

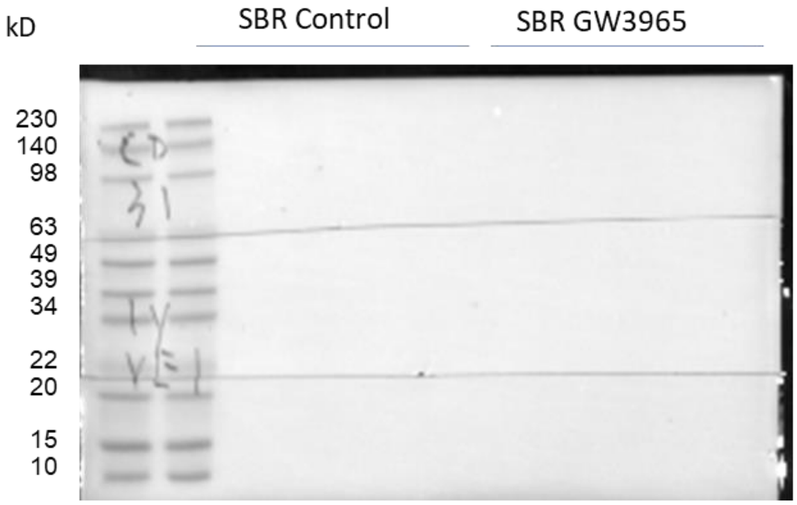
kD
SBR Control
SBR GW3965
230
140
98
63
49
39
34
22
20
15
10
